# Cardiac ryanodine receptor N-terminal region biosensors identify novel inhibitors *via* FRET-based high-throughput screening

**DOI:** 10.1016/j.jbc.2021.101412

**Published:** 2021-11-16

**Authors:** Jingyan Zhang, Daniel P. Singh, Christopher Y. Ko, Roman Nikolaienko, Siobhan M. Wong King Yuen, Jacob A. Schwarz, Levy M. Treinen, Ching-Chieh Tung, Kaja Rožman, Bengt Svensson, Courtney C. Aldrich, Aleksey V. Zima, David D. Thomas, Donald M. Bers, Bradley S. Launikonis, Filip Van Petegem, Razvan L. Cornea

**Affiliations:** 1 Department of Biochemistry, Molecular Biology and Biophysics, University of Minnesota, Minneapolis, Minnesota, USA; 2 School of Biomedical Sciences, The University of Queensland, Brisbane, Queensland, Australia; 3 Department of Pharmacology, University of California, Davis, California, USA; 4 Department of Cell and Molecular Physiology, Loyola University Chicago, Chicago, Illinois, USA; 5 Department of Biochemistry and Molecular Biology, Life Sciences Institute, University of British Columbia, Vancouver, British Columbia, Canada; 6 Department of Medicinal Chemistry, University of Minnesota, Minneapolis, Minnesota, USA

**Keywords:** ryanodine receptor, N-terminal region, FRET, fluorescence lifetime, high-throughput screening, myopathy, CaM, calmodulin, CPVT, catecholaminergic polymorphic ventricular tachycardia, EC, excitation–contraction, FA, fusidic acid, FKBP, FK506 binding protein 12.6, FLT, fluorescence lifetime, FWHM, full-width at half-maximum, HF, heart failure, HTS, high-throughput screening, NTR, N-terminal region, RyR, ryanodine receptor, SPR, surface plasmon resonance, SR, sarcoplasmic reticulum, WT, wild-type

## Abstract

The N-terminal region (NTR) of ryanodine receptor (RyR) channels is critical for the regulation of Ca^2+^ release during excitation–contraction (EC) coupling in muscle. The NTR hosts numerous mutations linked to skeletal (RyR1) and cardiac (RyR2) myopathies, highlighting its potential as a therapeutic target. Here, we constructed two biosensors by labeling the mouse RyR2 NTR at domains A, B, and C with FRET pairs. Using fluorescence lifetime (FLT) detection of intramolecular FRET signal, we developed high-throughput screening (HTS) assays with these biosensors to identify small-molecule RyR modulators. We then screened a small validation library and identified several hits. Hits with saturable FRET dose–response profiles and previously unreported effects on RyR were further tested using [^3^H]ryanodine binding to isolated sarcoplasmic reticulum vesicles to determine effects on intact RyR opening in its natural membrane. We identified three novel inhibitors of both RyR1 and RyR2 and two RyR1-selective inhibitors effective at nanomolar Ca^2+^. Two of these hits activated RyR1 only at micromolar Ca^2+^, highlighting them as potential enhancers of excitation–contraction coupling. To determine whether such hits can inhibit RyR leak in muscle, we further focused on one, an FDA-approved natural antibiotic, fusidic acid (FA). In skinned skeletal myofibers and permeabilized cardiomyocytes, FA inhibited RyR leak with no detrimental effect on skeletal myofiber excitation–contraction coupling. However, in intact cardiomyocytes, FA induced arrhythmogenic Ca^2+^ transients, a cautionary observation for a compound with an otherwise solid safety record. These results indicate that HTS campaigns using the NTR biosensor can identify compounds with therapeutic potential.

The ryanodine receptor (RyR), a homotetrameric (∼2.2 MDa) channel embedded in the sarcoplasmic reticulum (SR) membrane, is responsible for the robust Ca^2+^ release from SR storage to cytosol, to enable excitation–contraction (EC) coupling (1, 2). Defective RyR in skeletal (RyR1) or cardiac (RyR2) muscle cells perturbs Ca^2+^ release behavior, which can result in acquired and inherited myopathies (3, 4, 5, 6, 7, 8, 9). Therefore, RyRs are potential therapeutic targets for treating skeletal and cardiac myopathies, arrhythmias, and heart failure (HF) (9, 10, 11, 12, 13). Physiologically, the opening and closing of RyR channels are tightly regulated by small molecules, ions, and proteins (14), many of them binding to the enormous RyR cytoplasmic portion, with functional effects allosterically transduced to the cytoplasmic pore through long-range domain–domain interactions (15). The understanding of regulation by these domains has been greatly advanced by recent high-resolution cryo-EM RyR structures (15, 16, 17, 18, 19). The complex and rich regulation mechanisms of RyR domains offer opportunities to discover therapeutics that act either directly on RyR itself or modulate interacting proteins that determine RyR function.

Aiming to discover RyR regulators for therapeutic purposes, our group has previously developed a high-throughput screening (HTS) platform based on fluorescence-labeled RyR regulators FK506 binding protein 12.6 (FKBP) and calmodulin (CaM) to monitor binding of these modulators to RyR (20, 21). Using this assay with 1k-compound validation libraries, compounds have been identified to decrease RyR1 Ca^2+^ leak in skeletal muscle SR membrane vesicles and mechanically skinned muscle fibers (20, 21) and were further shown to mitigate force loss in a muscular dystrophy mouse model (22). However, the binding sites of these molecules are difficult to identify within the full-length RyR, which limits progress on understanding their mechanisms of action and hinders structure–activity relationship studies. Similar limitations reside with other recently reported HTS platforms for discovery using full-length of RyRs (23). An attractive solution to this problem would be to develop an HTS platform based on constructs with fluorescent proteins directly attached to the RyR (24). However, that is currently quite a challenging proposition for HTS applications, primarily due to the inadequate level of expression of the biosensor constructs. As a practical alternative, we are now exploring the use of an essential, crystallizable RyR fragment.

The N-terminal region (RyR residues 1–547; termed NTR) is composed of three domains (A, B, and C) and is important for the functional regulation of RyR channels (25). The regulatory role of the NTR is partially attributed to its structural location in the first tier of the cytosolic side of the RyR tetrameric channel complex (Fig. 1*A*) (17, 26). NTRs interact with each other through their respective domains A and B, forming a tetramer that delimits a vestibule leading to the RyR channel pore (Fig. 1*A*) (18, 27). The NTR also directly interfaces with the RyR bridging solenoid and then couples with the other cytoplasmic domains to allosterically regulate channel function (18). Very recently, it was proposed that NTR self-association is the “gatekeeper” of RyR2 channel activity. Specifically, a stable N-terminal tetramer maintains the RyR2 channel closure, whereas disruption of this tetramer results in channel dysfunction (28). In addition to structural evidence, the NTR is one of the three major clusters of mutations associated with skeletal and cardiac myopathies such as malignant hyperthermia (MH) and catecholaminergic polymorphic ventricular tachycardia (CPVT) (26, 27, 29, 30). Many of these disease-associated mutations in the NTR are found at the interfaces between domains A, B, and C. Some of these mutations reduce packing and destabilize the domain folding of the NTR, resulting in differences in structure or physical properties compared with the wild-type (WT) NTR. For example, CPVT-associated mutant R420Q-NTR lacks a Cl^−^ ion that has been observed at a central location of convergence among the three domains of WT-NTR. This Cl^−^ appears to confer RyR2 WT-NTR structural stability (27). Taken together, the NTR is a structurally and functionally important component of the RyR channels, and structural perturbation of the NTR has the potential to affect RyR channel activity.

**Figure 1:**
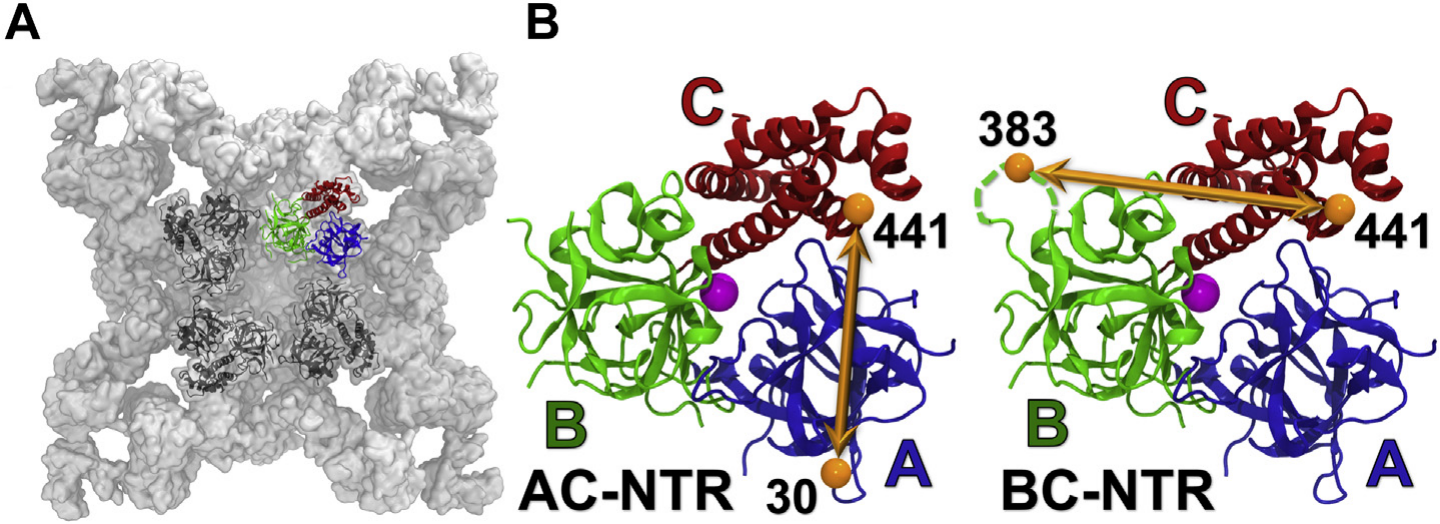
**Structural information on the NTR fragment of RyR2.***A*, location of the NTR within the cryo-EM RyR2 structure (PDB ID: 5GO9) (17). *B*, two FRET biosensors, AC-NTR and BC-NTR, were engineered using the NTR of mouse RyR2. For FRET labeling, Cys residues were substituted at K30 (domain A), R383 (domain B), or K441 (domain C) shown in the crystal structure of residues 1 to 547 of RyR2 (PDB ID: 4L4H) (26). *Dashed lines* represent the residues that are not resolved in the crystal structure. The *purple sphere* represents the chloride anion.

Given the critical role of the NTR in the regulation of RyR, we have engineered NTR constructs for use as FRET biosensors for an HTS platform to identify small molecules that interact with the NTR and allosterically regulate the activity of full-length RyR. We introduced donor–acceptor FRET pairs at appropriately distanced sites in different domains of the NTR (Fig. 1*B*), then used an FLT plate reader to measure changes in FLT-detected FRET efficiency as an indication of structural changes in the NTR due to small-molecule binding. Initial hit compounds obtained through FLT-FRET HTS of a small library were further validated using a dose–response format of the same FRET assay, and this was followed by functional assays (ryanodine-binding measurements) using full-length RyR in SR membrane vesicles of skeletal and cardiac muscles to further confirm their effects on RyR channels. The most promising hit was further evaluated on RyR leak in rat skeletal muscle fibers and mouse cardiomyocytes.

## Results

### Engineering FRET biosensor constructs based on the NTR of mouse RyR2

To build a FRET biosensor using isolated NTR, seven solvent-exposed Cys residues were first mutated into Ala, to obtain a Cys-light NTR. Per construct, two of three residues, K30 in domain A, R383 in domain B, and K441 in domain C, were then mutated to Cys, to provide the thiol labeling sites. Each test-biosensor construct contains two of the introduced Cys residues, K30C/K441C (termed AC-NTR), and R383C/K441C (termed BC-NTR). We used CD to evaluate the impact of mutations and labeling on the overall secondary structure of NTRs. As shown in Figure S1*A* (left panel), the similar CD spectra for WT and AC-NTR indicate that the Ala replacement of seven native Cys and addition of two Cys at K30 and K441 did not grossly alter the secondary structure of NTR. The BC-NTR has a marginally different CD spectrum relative to WT- and AC-NTR. CD spectra of the R420Q-NTR mutant and corresponding AC- and BC biosensor constructs also indicate that the engineered mutations did not alter the secondary structure content *versus* R420Q (Fig. S1*A*, right panel). However, the CD spectrum of R420Q-NTR shows a clear difference relative to WT-NTR. Previously, conformational changes had been observed for the R420Q mutant relative to WT (27). The CD experiments here suggest that this mutation may also lead to small changes in secondary structure content in solution. Based on the crystal structure shown in Figure 1, our chosen sites for Cys substitution (K30, R383, and K441) are fully exposed to the solvent and accessible for labeling using thiol-maleimide chemistry. We hypothesized that FRET-detected changes in their relative positions could provide an assay reflecting structural changes that occur in the NTR in response to ligand binding. The interprobe distances in the AC- and the BC-NTR constructs were estimated at 42 Å and 50 Å, respectively (note that R383 and K441 are not resolved in the crystal structure PDB ID 4L4H; instead, the nearby residues were used to estimate the interprobe distances; Fig. 1*B*) (27). Based on the predicted distances between the labeling sites in the two constructs, Alexa Fluor-488 and Alexa Fluor-568 fluorescent dyes were chosen as FRET donor and acceptor, respectively. Both constructs were labeled with a ratio of 2:3 between the donor and acceptor, to maximize FRET efficiency (Equation 1). Labeling also did not affect the WT- and R420Q-NTR secondary structure content, as determined by CD (Fig. S1*B*). Interprobe distances of the two biosensors were determined using previously described methods of multiexponential analysis of the FLT-detected FRET measurements (Fig. S2) (31) and were consistent with the distances predicted based on the crystal structure.

### Evaluation of the NTR FRET-biosensor sensitivity to small-molecule ligands

**Figure 2:**
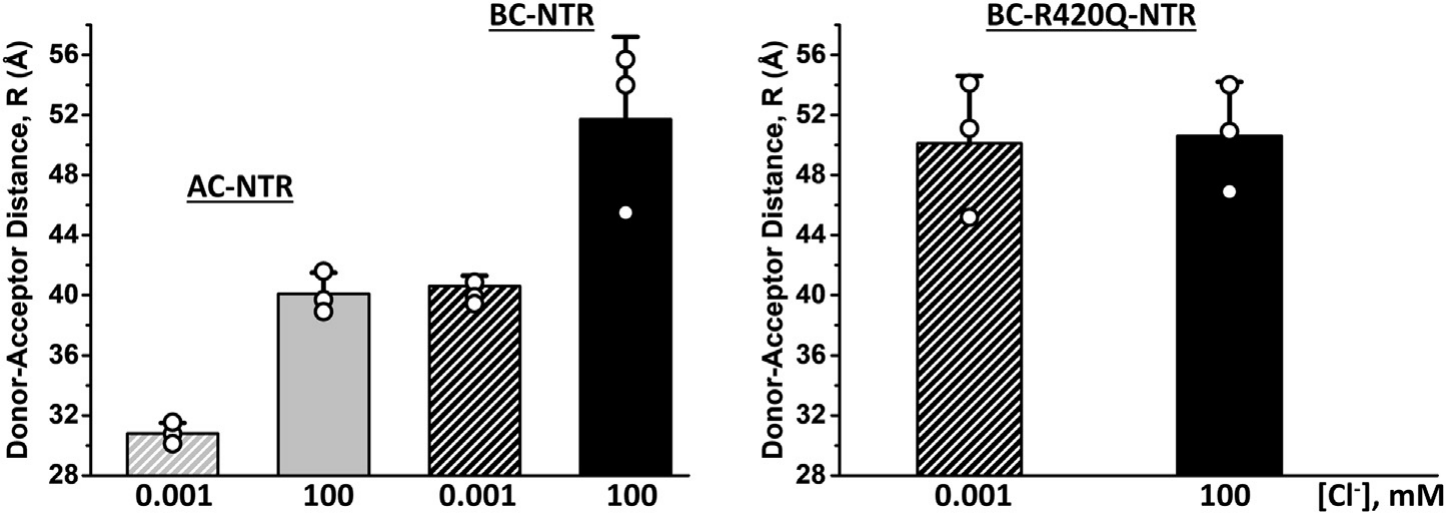
**Summary ofFRET-basedinter-probe (donor–acceptor) distances.** Cuvette-based FLT-FRET measurements were acquired via time-correlated single-photon counting, as described in Experimental Procedures. Inter-probe distances were determined for NTR and R420Q-NTR, within the AC- and BC-NTR constructs in the presence of low (0.001 mM) and high (100 mM) [Cl^−^] in the buffer, respectively (based on fitting parameters shown in Table S1 from the analysis of data such as shown in Fig. S2). Results are shown as mean ± SD (n = 3).

The AC-NTR and BC-NTR FRET constructs were evaluated by comparing the change in the inter-probe distances within NTR and R420Q-NTR determined *via* FRET data analysis in the presence of different concentrations of Cl^−^. The crystal structure of WT-NTR contains a Cl^−^ ion at a central position, stabilizing the protein through electrostatic interactions with nearby residues of R420, R298, and R276 (27). In practice, millimolar [Cl^−^] was necessary to stabilize NTR in solutions during the purification. However, Cl^−^ was not observed in the crystal structure of the R420Q-NTR mutant. We probed the reported structural difference between NTR and R420Q-NTR, and their responses to Cl^−^, to determine if the FRET assay of the two constructs (AC- and BC-NTR) is sensitive to small ligands. Multiexponential analysis of the FLT-detected FRET measurements was performed as previously described (32, 33, 34, 35). A single-Gaussian model, described by the interprobe distance (R) and its full-width at half-maximum (FWHM), provided the best fit of the AC- and BC-NTR and BC-R420Q-NTR FRET datasets, such as those illustratively shown in Figure S2. A summary of the FRET data fitting parameters is shown in Table S1, and Figure 2 illustrates R changes for these constructs in the presence of high (100 mM) and low (1 μM) [Cl^−^]. We observe ∼10 Å longer interprobe distances within NTR with both FRET-labeled constructs, in high [Cl^−^] *versus* low [Cl^−^] buffer, indicating that the two sensors can report ligand-induced changes in the NTR structure (Fig. 2, left). However, we observed no significant change in the interprobe distances of the R420Q-NTR mutant, in high *versus* low [Cl^−^] buffers (Fig. 2, right), indicating that the R420Q-NTR construct is insensitive to [Cl^−^]. This is consistent with Cl^−^ not being a ligand for R420Q-NTR (27). FWHMs of the R distributions were quite similar under both [Cl^−^] conditions, suggesting similar levels of disorder under the different [Cl^−^] conditions (Table S1). This result indicates that the tested AC- and BC-NTR constructs could be used as biosensors in studies to resolve small-molecule ligands of the NTR, such as done *via* HTS of chemical libraries containing drug-like molecules.

### FRET-based HTS using biosensor constructs derived from RyR2 NTR

FRET-labeled AC- and BC-NTR constructs were used to screen the 1280-compound LOPAC collection, which is a typical step for biosensor validation. HTS was carried out in 1536-well black-wall/black-bottom plates, formatted as described in our previous work (21, 36). A donor-only labeled construct was included for each screen as a control used to filter out the compounds that have a fluorescence signal overlapping with the donor fluorescence spectrum and which might therefore interfere with the FLT reading. Both the FLT waveforms and fluorescence spectra of each well in each plate were acquired after 20, 60, and 120 min of incubation with the LOPAC molecules. The FLT change of each well in the plate in the presence of LOPAC molecules was used as a high-precision assay of a structural change in the biosensor due to binding of a compound, previously shown to be ∼30-fold more precise than typically possible using fluorescence intensity (20). For a quantitative evaluation of the biosensors, we used the coefficient of variation (CV% = 100 × SD/means), which was 0.88 ± 0.13% for the AC construct and 1.41 ± 0.19% for the BC construct. Based on these CV values, a reference compound (positive control) would have to produce ΔFLT of at least 0.35 ns or 0.53 ns in order to achieve the “excellent” rating (*i.e.*, Z′ ≥ 0.5) for an assay driven by the AC or BC construct, respectively (per Equation 2) (37).

**Figure 3:**
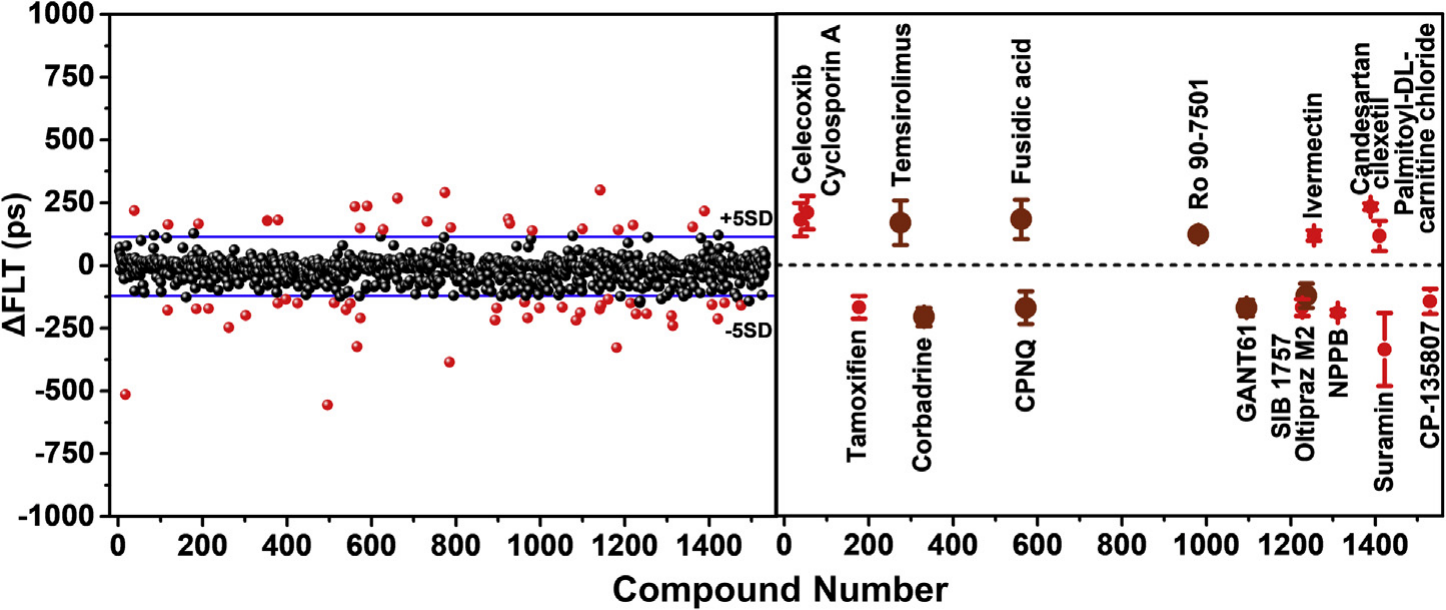
**Results from HTS of the LOPAC collection using NTR biosensors.***Left*: A representative FLT-based FRET HTS run with the labeled AC-NTR construct. *Bluelines* indicate the 5SD threshold. *Right*: 18 reproducible hits (*i.e.*, identified in ≥2 of 3 HTS runs) from AC- or BC-NTR constructs. These were further assessed using FRET dose–response measurements (Fig. 4). *Dark red* symbols indicate the hits selected for further testing. *Right panel* results are shown as mean ± SD (n = 3).

Library compounds that changed FLT beyond 5SD from the means of the DMSO controls were selected as hits. False-positive hits were filtered out based on changes in donor-only FLT, ratio between channel 1 and channel 2 intensity, fluorescence peak intensity and shape, and similarity index (38). Figure 3 (left panel) illustrates a representative outcome of the HTS. Each data point corresponds to the ΔFLT caused by one compound from LOPAC. Table S2 summarizes the Hit numbers and reproducibility at 20, 60, and 120 min incubation with the WT-AC and -BC constructs. Hit identities are indicated in Figure 3 (right panel). For each construct, these hits were found in at least two of the three runs of the screen, though longer incubation times resulted in more hits (Table S2 and Fig. S3). The screens using the AC-NTR construct picked up more hits than BC-NTR, as shown in Table S2. Some hits previously known to promote protein aggregation, as found in the Aggregator Advisor database (39), were not pursued in our next steps. Most of the remaining 18 hits are previously unreported RyR ligands (Fig. 3, right panel).

### FRET dose–response of the initial hits

To confirm the 18 initial hits, their FRET dose–responses were measured using the same method as in the primary HTS. In a range of 0 to 100 μM concentrations, the hits that decreased FLT in HTS also decreased FLT in this more detailed retest, and the same agreement was true for the hits that increased FLT. Compounds tamoxifen and CP-31398 decreased FLT of the donor-only sample by >50% of the FLT change in the donor–acceptor sample and therefore were designated interfering compounds and were excluded from further studies. Hits that showed ΔFLT <2% in these dose–response studies were also excluded from further evaluation. Based on these criteria, we narrowed down the number of hits for further evaluation to the eight compounds indicated by dark red symbols in Figure 3 (right panel). These are CPNQ, oltipraz M2, GANT61, corbadrine, fusidic acid (FA), tacrolimus, temsirolimus, and Ro 90-7501 (chemical names shown in Table S3).

**Figure 4:**
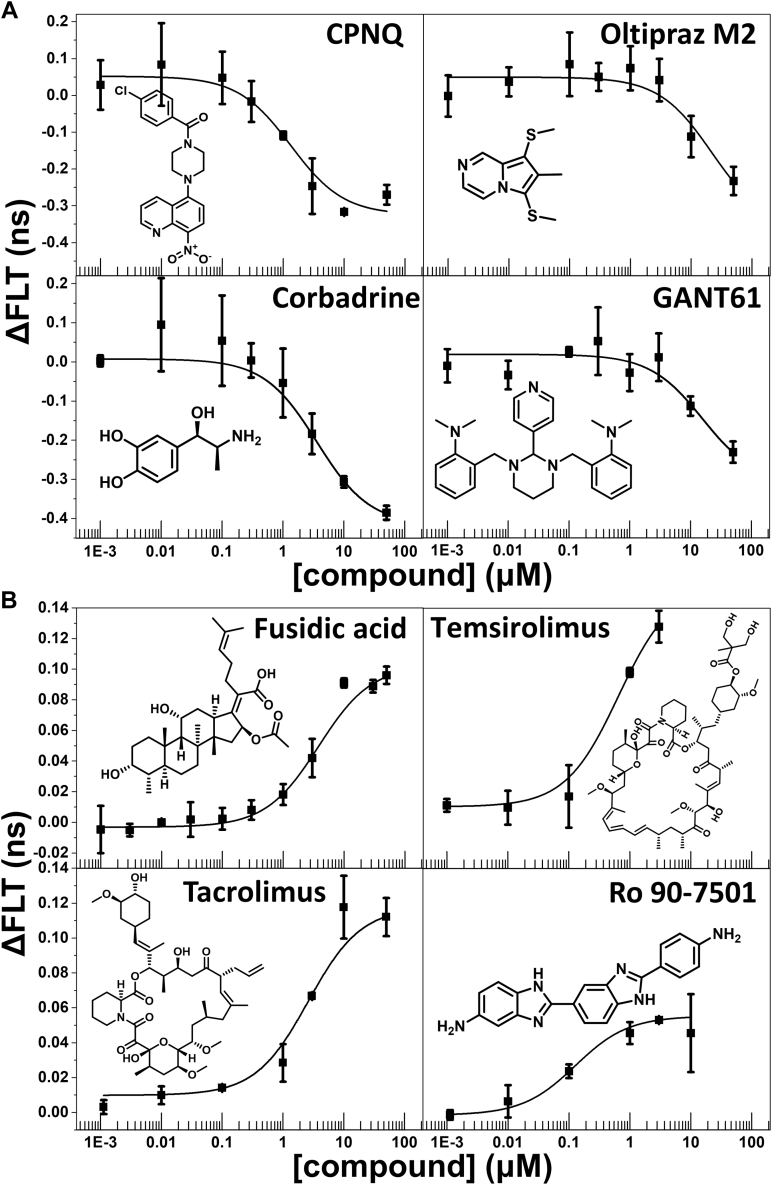
**FRET dose–response curves of the hits measured by the AC-NTR biosensor.** Compounds that decreased (*A*) and increased fluorescence lifetime (*B*) of the FRET biosensor. Plots are shown relative to the values for no-drug control (DMSO), mean ± SD (n ≥ 3).

Individual FRET dose–response curves of the eight remaining hits, along with their chemical structures, are shown in Figure 4. The solid lines represent fits of the experimental data using the Hill function. Among the hits that decreased FLT (Fig. 4*A*), CPNQ had the strongest effect (ΔFLT ≈0.35 ns), which was similar to corbadrine, albeit the latter had lower potency (∼3-fold higher EC_50_). Both GANT61 and oltipraz M2 decreased FLT but did not reach saturation within the tested concentration range, indicating a lower potency compared with corbadrine and CPNQ. Among the hits that increased FLT (Fig. 4*B*), FA and tacrolimus exhibited a stronger effect than the other two compounds in this group (ΔFLT ≈0.1 ns). Temsirolimus and Ro 90-7501 produced smaller increases in FLT, but with a higher potency (EC_50_ <1 μM). Temsirolimus and the related tacrolimus (aka FK506) are known ligands of FKBP, and they belong to a group of compounds thought to indirectly influence the RyR function, by preventing FKBP-RyR binding (40). Our results (Fig. 4*B*) indicate that these compounds may also directly interact with the RyRs, a hypothesis to be explored in future studies. Tacrolimus and Ro 90-7501 had already been identified as RyR regulators in our previous HTS campaigns (*via* a FRET HTS assay that uses full-length RyRs), and their RyR activatory and inhibitory effects (respectively) have been reported (20, 21). The remaining five compounds are previously unknown RyRs ligands, and we further evaluated them by [^3^H]ryanodine-binding assays (shown below).

### Effect of hits on [^3^H]ryanodine binding to full-length RyR1 and RyR2

To functionally evaluate the hits that had a clear FRET dose–response profile and previously unreported effects on RyR function, we performed [^3^H]ryanodine-binding assays using SR membrane vesicles isolated from pig skeletal and cardiac muscle. This is a well-established method that is often used to determine the activity of RyR channel in their native membrane environment (41) and is therefore routinely used to evaluate hit effects on RyRs (20, 21, 23). The increase or decrease of bound [^3^H]ryanodine correlates with the fractional population of RyR open channels. [^3^H]ryanodine-binding assays were carried out in the presence of low Ca^2+^ (30 nM) and high Ca^2+^ (30 μM), corresponding to the resting and contracting muscle conditions, respectively. This is to glean early insight in the potential effect of a hit on the resting RyR leak, as well as on RyR activation during EC coupling.

**Figure 5:**
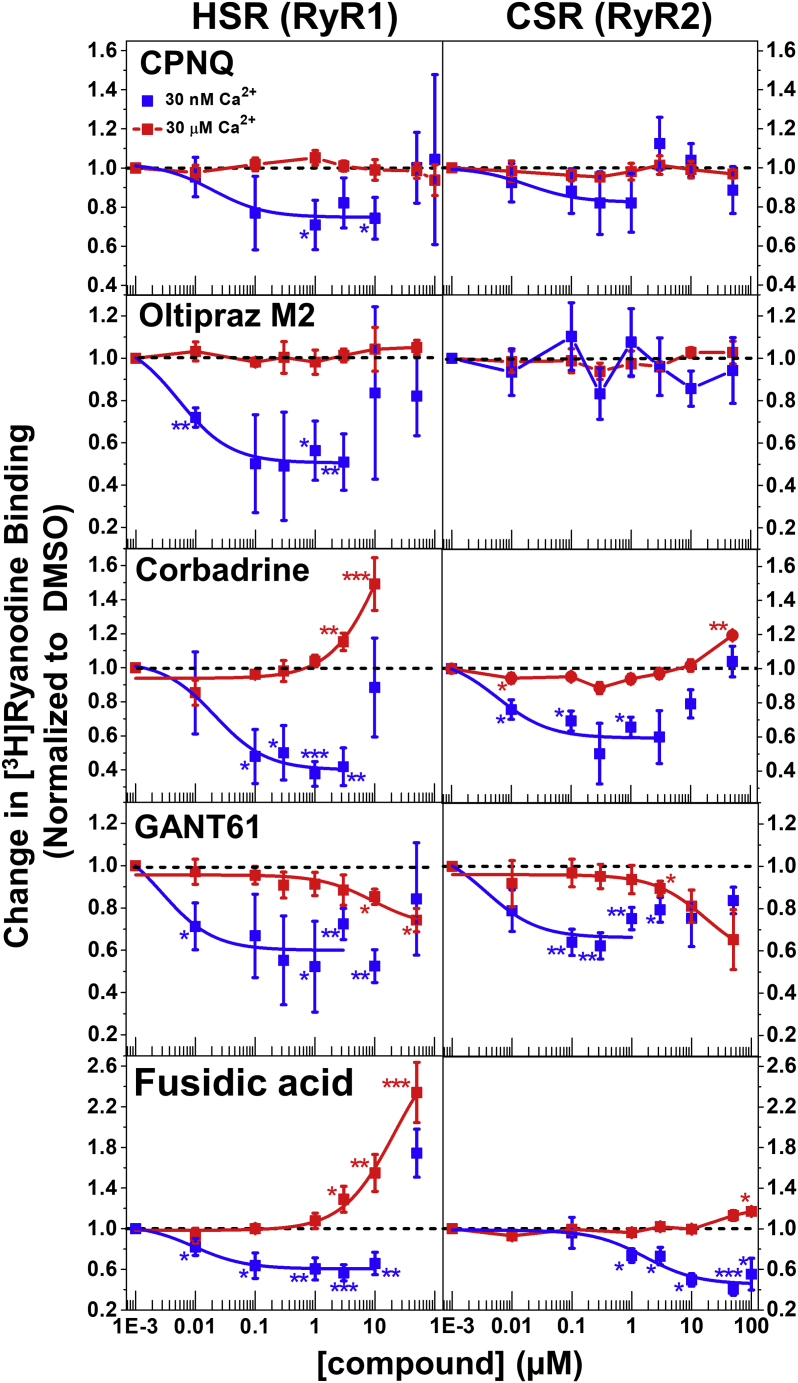
**Dose-dependent effects of the hits on [**^**3**^**H]ryanodine binding to skeletal SR.** Experiments were carried out using skeletal SR (HSR, *left*) and cardiac SR (CSR, *right*) vesicles, at 30 nM (*blue*) and 30 μM (*red*) free Ca^2+^. Results are shown normalized relative to the values for no-drug control (DMSO), respectively, mean ± SD, n ≥ 4. Data were fit using the Hill function. Due to the biphasic shape of the dose–response, the points in the ascending portion of the profiles were excluded from fitting. This biphasic behavior is often seen in pharmacology and can be caused by multiple binding sites of the target, nonspecific modulation of the target caused by colloidal aggregation of ligands at high concentrations, and off-target activity. The *asterisks* (∗,∗∗,∗∗∗) indicate the level of significance in the difference from DMSO controls using unpaired Student’s *t* test: ∗∗∗*p* ≤ 0.001 (extremely significant), 0.001 ≤ ∗∗*p* < 0.01 (very significant), 0.01 ≤ ∗*p* < 0.05 (significant), no-asterisk *p* ≥ 0.05 (statistically insignificant difference relative to the DMSO control).

RyR1 and RyR2 share more than 65% sequence similarity, and the NTRs of RyR1 and RyR2 also highly resemble each other in terms of sequence and structure (42). Thus, modulators of one isoform might also affect the other. Therefore, we performed [^3^H]ryanodine-binding analyses to determine the dose-dependent effects of the novel FRET hits (from Fig. 4) on RyR1 and RyR2 function, in skeletal (Fig. 5, left) and cardiac membrane vesicles (Fig. 5, right), respectively.

For skeletal SR (RyR1) at 30 nM Ca^2+^ (Fig. 5, left panels, blue curves), all tested hits inhibited [^3^H]ryanodine binding in a concentration-dependent manner, with nanomolar IC_50_ values (as indicated). All discussed compounds show biphasic responses, with inhibition by submicromolar concentration gradually turning into activation at the highest concentrations tested (Figure 5, left; Fig. S4). Maximum inhibitory effects observed range from approximately 30% for CPNQ, to 50% for oltipraz M2, GANT61, and FA, to 70% for corbadrine (Figure 5, left; Fig. S4). The biphasic dose–response profile (hormesis) is often observed in biological systems and particularly in pharmacology (43). This may have an underlying mechanistic basis caused by multiple modes of binding to the target (*e.g.*, to high and low affinity sites) or by nonspecific inhibition caused by colloidal aggregation of ligands at high concentrations (44, 45), among other reasons. In particular, SR vesicles represent a heterogeneous sample (*i.e.*, they are not fully purified RyRs), meaning that these dose–response profiles may represent RyRs that are not in complex with the same set of binders or carrying different posttranslational modifications.

For skeletal SR at 30 μM Ca^2+^ (Fig. 5, left panel, red curves), both CPNQ and oltipraz M2 only slightly affected the [^3^H]ryanodine binding to RyR1 throughout the tested concentration range, and GANT61 showed a gradual inhibition at concentrations >1 μM. However, concentrations >1 μM of corbadrine and FA strongly increased [^3^H]ryanodine binding.

For cardiac SR (RyR2) at 30 nM Ca^2+^ (Fig. 5, right panel, blue curves), the pattern of inhibitory effects by four of the hits was largely similar to RyR1. Oltipraz M2 was the exception, as it had almost no effect on RyR2. Inhibition of RyR2 by FA had micromolar IC_50_ (3.7 μM) *versus* nanomolar (0.03 μM) for RyR1.

For cardiac SR at 30 μM Ca^2+^ (Fig. 5, right panel, red curves), CPNQ and oltipraz M2 did not significantly alter [^3^H]ryanodine binding, similar to HSR. Corbadrine and FA showed slight activation at high concentrations, but much smaller than with HSR. GANT61 was an inhibitor at concentration >1 μM, similar to HSR.

### Focusing on FA for therapeutic repurposing

Taken together, our FRET (Fig. 4) and [^3^H]ryanodine binding (Fig. 5) assays and previously reported favorable pharmacologic properties indicated that FA is a drug with potential for repurposing as therapeutics targeting leaky RyRs. Therefore, we focused more detailed studies in support of this goal. First, we performed surface plasmon resonance (SPR) measurements to confirm *via* this label-free method that FA binds specifically to the NTR. SPR data indicates that the compound binds with low micromolar K_D_ to the NTR (with a His-MBP tag for immobilization on the SPR sensor chip) (Fig. S5). This is consistent with the EC_50_ measured using the same FRET assay as in the primary HTS (Fig. 4, Table S4).

### Effect of FA on RyR1 leak and EC coupling in rat skeletal muscle skinned fibers

We explored the effects of FA on RyR1 in a physiological system with intact EC coupling apparatus by measuring SR Ca^2+^ leak using a confocal microscopy readout of enclosed transverse-tubule [Ca^2+^] in rat mechanically skinned skeletal muscle fibers (46).

**Figure 6:**
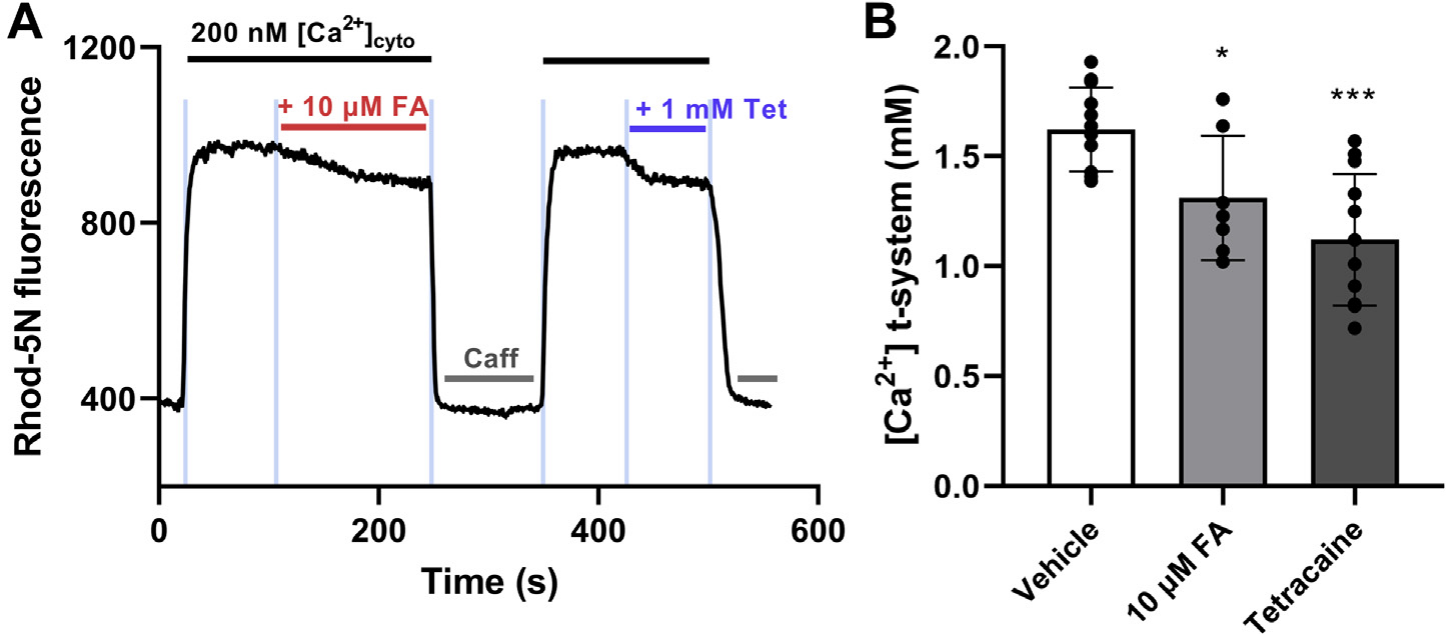
**Effect of fusidic acid (FA) on t-tubule Ca**^**2+**^**measurements of RyR1 leak in rat skinned skeletal muscle fibers.***A*, representative raw fluorescence trace of t-tubule Ca^2+^ in response to 10 μM FA and 1 mM tetracaine. *B*, bar graph displaying normal t-system Ca^2+^ uptake and full RyR activity (vehicle), reduced t-system [Ca^2+^] by 10 μM FA (reduced RyR Ca^2+^ leak) and full RyR inhibition by tetracaine. A one-way ANOVA with Dunnett’s multiple comparisons post-hoc test was performed (F = 11.53, *p* = 0.0002). The multiple comparisons test showed significance between vehicle *versus* 10 μM FA (*p* = 0.0315) and vehicle *versus* 1 mM tetracaine (*p* = 0.0001). Data presented as means  ±  SD, ∗*p* < 0.05, ∗∗∗*p* < 0.001 *versus* vehicle (DMSO), n  =  7 to 12 individual fibers.

As described in previously published work, in a typical experiment (47), the conditions resemble those observed in many myopathies, where [Ca^2+^]_cyto_ is maintained at 200 nM, which is above resting physiological levels, thus overloading the SR and inducing an increase of RyR Ca^2+^ leak. Rhod-5N fluorescence was used to monitor [Ca^2+^]_t-sys_ before and after bath exchange with one concentration of FA or tetracaine. To determine the total [Ca^2+^]_t-sys_ and the contribution of RyR1 leak to [Ca^2+^]_t-sys_, the bath solution with 30 mM caffeine (an RyR activator) and 1 mM tetracaine (an RyR blocker) were exchanged, respectively. As shown in Figure 6*A*, in the presence of 10 μM FA in bath solution, the steady-state [Ca^2+^]_t-sys_ decreased, indicating inhibition of RyR1 leak by FA. The recovery of the t-system fluorescence after washout with caffeine and reintroduction of standard solution indicates that FA can be washed out of the fiber. Figure 6*B* compared normal t-system Ca^2+^ uptake and t-system [Ca^2+^] that reduced [Ca^2+^] by 10 μM FA and tetracaine. This suggests that high FA inhibits RyR1 leak.

In addition to the effect of FA on RyR1 leak, we also explored its potential influence on EC coupling in rat skeletal muscle (Fig. S6). Electrically induced Ca^2+^ transients were generated in single rat muscle fibers in the presence of DMSO (0.1%), 10 μM FA, and 1 mM tetracaine. Interestingly, 10 μM FA did not have an effect on Ca^2+^ transient amplitude. As a comparison the application of tetracaine, which is a full RyR blocker, significantly decreased the electrically induced Ca^2+^ events. Ideally, therapeutically targeting and reducing excessive RyR1 Ca^2+^ leak should not be accompanied with a reduction in normal muscle contraction. These results show that FA can reduce RyR1-mediated Ca^2+^ leak without a significant effect on normal EC coupling.

### Effect on Ca^2+^ sparks and transients in mouse ventricular cardiomyocytes

**Figure 7:**
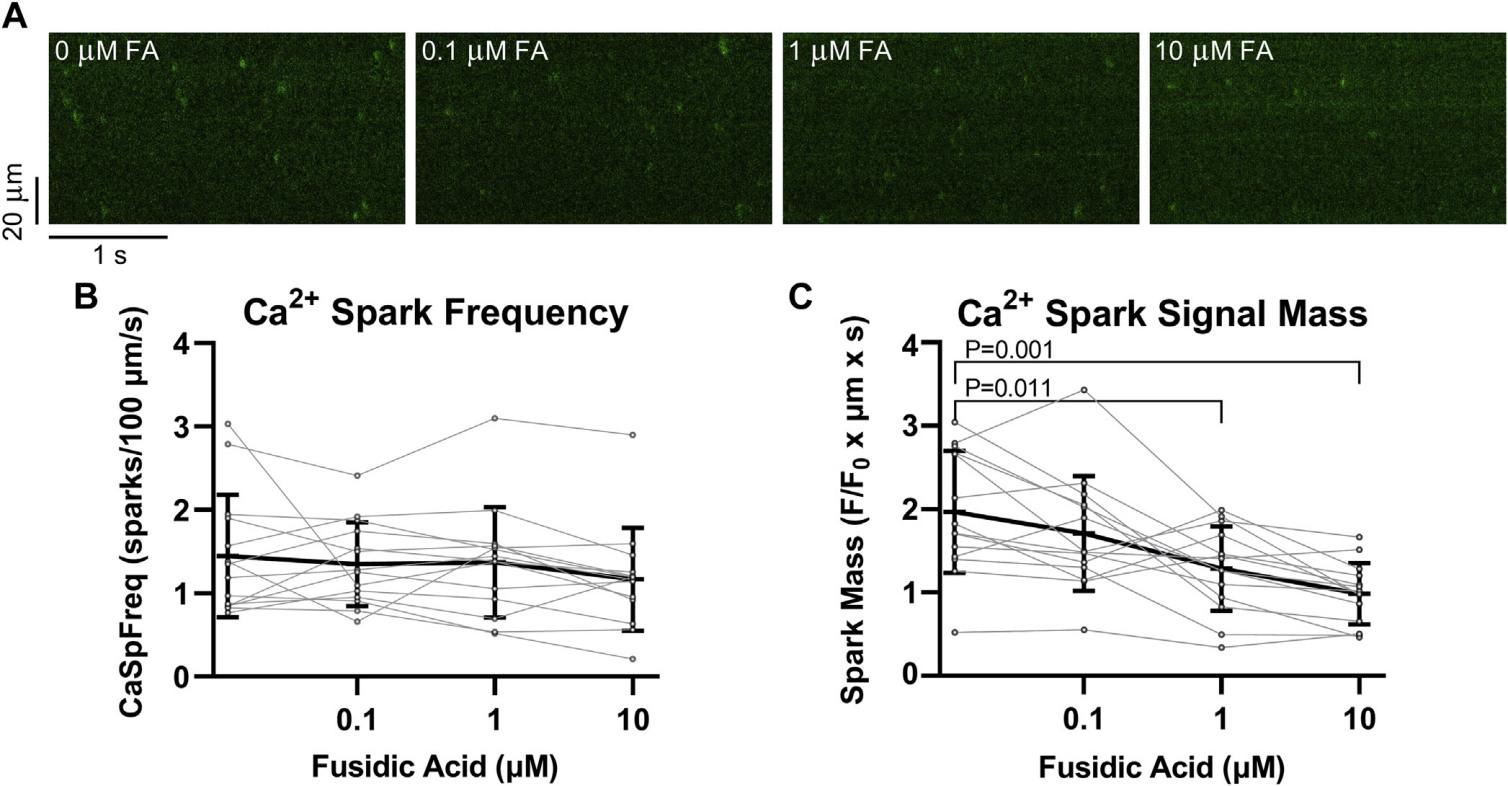
**Effect of fusidic acid (FA) on RyR2-mediated Ca**^**2+**^**leak in mouse ventricular cardiomyocytes.***A*, representative line scan recordings of Ca^2+^ spark activity in a permeabilized mouse ventricular cardiomyocyte after exposure to increasing concentrations of FA (6 min each). Free [Ca^2+^]_i_ = 50 nM. *B*, Ca^2+^ spark frequency (CaSpFreq) (mean_cell_ ± SD) responses to increasing concentrations of FA. n_cell_/N_animal_ = 14/3. Friedman one-way ANOVA (*p* = 0.277, Friedman statistic = 3.857); Dunn’s multiple comparison post-hoc test, relative to 0 μM FA. Mean_cell_ slope = −0.082 sparks/100 μm/s/log_10_(μM)); one-sample *t* test of individual slopes (*p* = 0.115). *C*, Ca^2+^ spark signal mass (amplitude × FWHM × full-duration at half maximum) (mean_cell_ ± SD) response to increasing [FA]. Matched n_cell_/N_animal_ = 14/3. Repeated measures one-way ANOVA with Geisser–Greenhouse correction (*p* = 0.0004, F = 12.27); Dunnett’s multiple comparison post-hoc test, relative to 0 μM FA (*p* = 0.011 for 1 μM FA; *p* = 0.001 for 10 μM FA). Mean_cell_ slope = −0.336 F/F_0_ × μm × s/log_10_(μM)); one-sample *t* test of individual slopes (*p* = 0.0007).

We first used saponin-permeabilized mouse ventricular cardiomyocytes (at [Ca^2+^]_i_ = 50 nM) to assess Ca^2+^ spark activity, a relatively direct measure of SR Ca^2+^ leak, during 6 min exposures to increasing [FA] (Fig. 7, *A*–*C*). In repeated measures of Ca^2+^ spark frequency over [FA], we did not detect significant differences between [FA], relative to 0 nM FA (*p* = 0.277, Friedman statistic = 3.857), though the data suggest a possible minor trend for reduced Ca^2+^ spark frequency (mean_cell_ slope = −0.082 sparks/100 μm/s/log_10_(μM)) with increasing [FA] (*p* = 0.115) (Fig. 7*B*). However, repeated measures of Ca^2+^ spark signal mass (amplitude × FWHM × full-duration at half maximum), a readout of the amount of Ca^2+^-spark mediated SR Ca leak, was decreased significantly at 1 μM FA (*p* = 0.011) and 10 μM FA (*p* = 0.001), relative to 0 nM FA. Moreover, Ca^2+^ spark signal mass exhibited a progressively decreasing trend (mean_cell_ slope = −0.336 F/F_0_ × μm × s/log_10_(μM)) with increasing [FA] (*p* = 0.0007), indicating progressively smaller release events (Fig. 7*C*). Together, the Ca^2+^ spark data suggest that FA suppresses SR Ca^2+^ leak in a dose-dependent manner primarily by limiting SR Ca^2+^ release during individual events.

We also measured Ca^2+^ transients in intact mouse ventricular cardiomyocytes during pacing at 1 Hz. In most cardiomyocytes, even at very low [FA] (0.01 μM), we observed some diminution of Ca^2+^ transient amplitudes and an increased occurrence of arrhythmogenic Ca^2+^ activity (Fig. S7*A*). These Ca^2+^ events appeared as isolated spontaneous Ca^2+^ transients or runs of automaticity in the absence of pacing, Ca^2+^ waves, or persistent diastolic Ca^2+^ elevation (Fig. S7, *A* and *B*). These events were almost never seen in control conditions (1 of 12 total paired measures) but were seen in the majority of cardiomyocytes (10 of 12 total paired measures) exposed to FA across all concentrations tested (0.01–10 μM). Thus, while FA may indeed have a quieting effect on RyR2, as inferred from the Ca spark measurements, it may also have side effects that increase myocyte Ca^2+^ loading or otherwise promote automaticity.

### Effect of FA on ER Ca-load in HEK293 cells

The myocyte results are consistent with the FA effects on ER Ca-load [Ca^2+^]_ER_ observed in HEK cells expressing human RyR2 (Fig. S8), as previously described (48). These measurements were carried out using permeabilized HEK293 cells coexpressing GFP-hRyR2, mCer-SERCA2a, along with R-CEPIA1er, which is a fluorescent biosensor of the ER Ca^2+^ concentration. [Ca^2+^]_ER_ increased 94 ± 17% upon treatment with 5 μM FA, as expected when the Ca leak through the resting channel is inhibited. We also observed a [Ca^2+^]_ER_ decrease of 24 ± 8% upon increasing FA to 10 μM, which might be a result of the biphasic effect illustrated in Figure S4.

## Discussion

RyR channels are under intense study as potential therapeutic targets for diseases ranging from rare (but extremely diverse) ryanopathies to high-prevalence degenerative syndromes. Here, we used the FRET-labeled NTR to detect compound binding, as this is a domain that is critically involved in RyR function. Specifically, NTR assembles into a tetrameric vestibule (Fig. 1*A*) that is sterically coupled to the channel pore regulation. The NTR is also one of the “hot-spots” of disease-associated mutations of RyR (26, 27, 29). Structural perturbation of the NTR itself or of its interaction with adjacent domains leads to disruption to the open/closed properties of RyR channels. For example, in the related IP3 Receptor, the corresponding NTR forms the binding site for the activating ligand IP3 (7, 49), which alters the conformation of the NTR. Therefore, small-molecule ligands of the NTR in RyRs have the potential to functionally regulate the full-length RyR channels and be developed *via* medicinal chemistry aided by crystallography and functional studies to obtain therapeutic agents against RyR dysfunction.

To exploit the unique structural features and functional importance of the NTR, and the well-established high-precision FLT detection of FRET, we constructed two NTR biosensors by substituting Cys at two residues located in A and C, or B and C subdomains of the Cys-light NTR, and labeled these Cys pairs with fluorescent probes suitable as FRET donor and acceptor (Fig. 1). The two NTR biosensors with the fluorescent probes located at different domains are expected to be sensitive to different structural changes and, therefore, detect different modulators of the NTR. Indeed, different interprobe distances are reported by the two biosensors in response to chloride ions, validating that the constructed biosensors are responsive to changes in NTR structure/conformation (Fig. 2) and suggesting that these constructs could be used in FRET-based HTS assays for RyR-targeted drug discovery. These results also support the hypothesis that binding or unbinding of chloride to the RyR2 NTR may regulate channel activity (27).

Using these two biosensor constructs in FRET-based HTS of the LOPAC chemical collection, we identified hits that either increased or decreased the FLT of the biosensors (Fig. 3). Screening with AC-NTR picked up more hits than BC-NTR, possibly due to the probe at K30 in the A domain being more sensitive than R383 in the B domain. Also, more hits decreased FLT than increased it (Fig. S3). Based on reproducibility and chemical and physical properties, we selected 18 initial hits (Fig. 3*B*) for further testing. The list of hits was further narrowed down to eight compounds based on FRET dose–response curves (Fig. 4) and compound availability. Among the eight compounds, three were previously identified as potential RyR regulators, leaving five newly identified compounds for testing *via* assays of [^3^H]ryanodine binding to full-length RyR1 and RyR2 (Fig. 5).

Five of the eight identified hits are structurally and functionally different from known RyR modulators, such as 1,4-benzothiazepine derivatives (aka rycals, *e.g.*, JTV519 and S107), or dantrolene (50, 51, 52). These five hits are also structurally unrelated to the RyR small-molecule modulators discovered using biosensor systems using labeled FKBP and CaM (21). Of the five validated hits, four shortened the FLT (increased FRET), suggesting a shorter interprobe distance within NTR; and one lengthened FLT (decreased FRET), suggesting a longer interprobe distance. All five FLT hits also affected [^3^H]ryanodine binding to RyR1 and RyR2, which provides experimental support to the hypothesis that NTR ligands can regulate the functional status of RyR channels, as suggested by high-resolution structures of RyR channels and many functional studies (17, 26, 28). Although the FRET biosensor has been developed based on the RyR2-NTR, there is a high degree of homology with the RyR1-NTR (∼70% sequence identity). Therefore, it is not surprising that the Hits interact and functionally modulate both full-length RyR isoforms, often with surprising results. In general, this is to be expected when protein modifications are introduced to engineer biosensors for HTS assays. Isoform specificity is more effectively established through secondary (downstream) assays, orthogonal to the primary HTS assay.

Discrepancies in IC_50_ values obtained from FRET and [^3^H]ryanodine-binding assay are notable but not unexpected. This is because the FRET structural measurements and the functional assays are strongly driven by the nature of the samples, *i.e.*, isolated soluble fragment of RyR *versus* full-length RyR in isolated SR vesicles, or *versus* cellular environment.

Among the five hits, oltipraz M2 exhibited strong isoform specificity by inhibiting RyR1 only in nanomolar [Ca^2+^]. Oltipraz M2 is a metabolite of cancer chemopreventive agent oltipraz. It has been shown to exert cytoprotective effects *via* antioxidation mechanisms (53, 54). CPNQ targets huntingtin and α-synuclein to lower their pathological effects in cellular models of Huntington’s and Parkinson’s diseases, respectively (55), and we show here that this compound significantly inhibited only RyR1 (with an inhibitory trend on RyR2) at low [Ca^2+^]. GANT61, a hexahydropyrimidine derivative antitumor agent (56), is the only Hit that inhibited RyR1 and RyR2 channels at both low and high [Ca^2+^].

Corbadrine and FA affected [^3^H]ryanodine binding to RyR1 and 2 most strongly among the tested compounds (Fig. 5). Thus, the [^3^H]ryanodine-binding profiles in the presence of corbadrine and FA—in nanomolar *versus* micromolar Ca^2+^—are particularly attractive for candidate RyR1 therapeutics. However, corbadrine has potentially significant liabilities due to its adrenaline-analogous structure and the very high binding affinity for α2-adrenergic receptors, suppressing peripheral vasoconstriction (57). Also corbadrine has a high similarity to the known aggregator norephedrine (58). For these reasons, corbadrine might be unsuitable for repurposing, despite its favorable effects on RyR1 and RyR2 in isolated SR membranes.

On the other hand, FA has a good safety record and excellent pharmacokinetic profile, and good tissue distribution (59), thus showing potential as a lead compound. Its reversible inhibitory effect on SR Ca^2+^ leak in rat skinned skeletal muscle fibers suggests that FA affects RyR1 even when the EC coupling apparatus is intact (Fig. 6). Furthermore, the overall FA inhibition of SR Ca^2+^ leak observed in permeabilized cardiomyocytes also suggests a direct effect on RyR2 (Fig. 7).

A viable candidate for a myopathy therapeutic application would be expected to have no detrimental effect on EC coupling. Therefore, we were encouraged to observe unchanged electrically evoked Ca^2+^ transients in skeletal muscle fibers (Fig. S6). However, similar measurements in cardiomyocytes revealed increased arrhythmogenic activity (Fig. S7), although the molecular mechanism was not identified. In light of FA’s good safety record (59), we were surprised to discover these potentially deleterious effects on isolated cardiomyocytes. These highlight the need for further studies to understand the molecular basis of FA’s effect on muscle Ca^2+^ fluxes.

FA is an FDA-approved bacteriostatic antibiotic derived from the fungus *Fusidium coccineum* and is used as a topical medication to treat skin infections by inhibiting bacterial protein synthesis (60). Although FA is rarely used as an antibiotic in the United States because of drug resistance, it has a good safety profile and is well tolerated. As it is often the case with antibiotics, FA is an attractive candidate for repurposing (59). Derivatives of FA have been synthesized (61), and they could be tested in future studies focused on establishing structure–activity relationships relevant to RyRs.

Among the previously identified RyR regulators (20, 21), tacrolimus and temsirolimus are known FKBP ligands thought to prevent FKBP binding to RyRs. Tacrolimus was also identified by HTS using a measurement of endoplasmic reticulum Ca^2+^ in HEK293 cells (23). Given that tacrolimus and temsirolimus are known high-affinity ligands of FKBP, it was somewhat unexpected to identify them as ligands of our NTR biosensors. This observation suggests that they have the potential to also interact directly with full-length RyR (15, 62).

In summary, we developed an early-stage RyR-targeted drug discovery method exploiting high-throughput FLT-detection of FRET within an RyR2 NTR construct labeled with donor and acceptor, to identify small-molecule *allosteric* modulators of RyR1 and RyR2. We identified several novel RyR1 and RyR2 effectors. Results from the *in vitro* biochemical assays suggest that none of these compounds have deleterious effects on RyR1 and RyR2, and some (*e.g.*, oltipraz M2 and FA) should be further pursued through studies using cell and animal models of skeletal and cardiac myopathies. *In situ*, cellular assays of RyR leak reinforce the *in vitro* results, as shown before. However, potentially deleterious effects with intact cardiomyocytes indicate that further safety studies are necessary for FA. Further studies will be necessary to determine the mechanisms of action of these new effectors and pursue medicinal chemistry development toward an RyR-targeted drug. Notable limitations of the NTR biosensor are typical to target-focused approaches, whereby a particular biosensor system can never identify all the ligands that may interact with the target, or that the Hit functional effects are often unpredictable. Nevertheless, we are encouraged about the outcomes of this study and plan future HTS screening of the NTR biosensor against a larger chemical library, to accelerate the discovery of RyR-targeted therapeutics.

## Experimental procedures

### Expression, purification, and labeling of NTR

The NTR of mouse RyR2 (residues 1–547) was cloned and expressed as previously described (27). The DNA construct contains a 6×His tag, a maltose-binding protein (MBP) tag, and a tobacco etch virus (TEV) protease cleavage site. The seven native solvent-exposed cysteine residues were substituted with alanine residues, and two constructs were engineered with Cys substitutions at surface residues R383 and K441 (in the B and C domains, respectively; termed BC) and at K30 and K441 (in the A and C domains, respectively; termed AC) for labeling with spectroscopic probes such as appropriate donor–acceptor fluorophores for FRET (for the purpose of this report, these are considered the WT forms of AC and BC). A variant of the BC construct corresponding to the disease-associated mutant (R420Q) was also prepared. For this site-directed mutagenesis work, we used the QuikChange (Stratagene) and Phusion High-Fidelity PCR (Thermo Fisher Scientific) kits. The WT-NTR, R420Q-NTR, AC- and BC-NTR, AC- and BC-R420Q-NTR protein constructs were expressed in *Escherichia coli* Rosetta (DE3) pLacI cells at 18 °C and purified with a protocol adapted from previously published work (26). An amylose column was first applied after the cells were lysed by ultrasonication. The protein in the elution was then cleaved using TEV protease. The cleaved crude protein went through the second amylose column to separate from the MBP and then followed by a Superdex S-200 size exclusion column in a buffer consisting of 250 mM KCl, 10 mM HEPES, and 14 mM β-mercaptoethanol (pH 7.4). The NTR protein (3 μM) was then incubated in 10 mM HEPES, 250 mM KCl (pH 7.4, 2 h, 21 °C) with the fluorescent probes Alexa Fluor 488 maleimide (AF488, donor; 40 μM) and Alexa Fluor 568 maleimide (AF568, acceptor; 60 μM). The maleimide of the dye reacts with the thiols the Cys residues engineered into NTR, forming a covalently labeled protein. The Alexa dyes were added last to the reaction mixture, from 10 mM DMSO stock solutions. This reaction is not selective to one site (K30C or K441C), and we anticipate that there is a mixed population with K30C or K441C labeled with either AF488 (donor) or AF568 (acceptor). We focused on maximizing the fraction containing donor–acceptor pairs, thus maximizing FRET efficiency and the precision for detecting a change in structure as an indication that a compound interacts with the NTR biosensor. The labeling efficiency was determined by comparing the molar concentration of the bound dye, determined from UV-Vis absorbance, to the protein concentration, determined from gel densitometry. Essentially complete labeling was confirmed from electrospray ionization mass spectrometry. CD spectra of the WT-NTR and mutant R420Q-NTR constructs before and after labeling were recorded using a JASCO J-815 CD spectrometer (JASCO), confirming similar folding (Fig. S1).

### Chemical library handling, preparation of 1536-well assay plates and sample loading

The 1536-well assay plates were prepared as described in our previous reports (21). The LOPAC (Sigma-Aldrich) collection received in 96-well plates was reformatted into 384-well polypropylene intermediate plates (Greiner Bio-One) using a multichannel liquid handler, BioMek FX (Beckman Coulter), then transferred to 384-well Echo Qualified source plates (Labcyte Inc). Assay plates were prepared as previously described (20). On screening days, these plates were thawed out and were loaded with labeled NTR (5 μl, 20 nM final concentration) using a Multidrop Combi automated sample dispenser.

### FRET measurements

For cuvette-based determinations of NTR construct response to Cl^−^, time-correlated single-photon counting measurement of fluorescence lifetime (FLT) was used. Time-resolved fluorescence decays were recorded after excitation with a 481-nm subnanosecond pulsed diode laser (LDH-P-C-485, PicoQuant). Emitted light was selected using a 517 ± 10 nm filter (Semrock) and detected with a PMH-100 photomultiplier (Becker-Hickl). FLT waveforms of the donor only and donor–acceptor-labeled samples were acquired in media containing 60 nM biosensor, 10 mM HEPES, 250 mM KCl, pH 7.4 at 21 °C and analyzed to determine FRET distance relationships, as described in our previous publications (32, 33, 34, 35).

HTS measurements were conducted in a prototype top-read FLT plate reader (FLT-PR, Fluorescence Innovations, Inc), which reads each 1536-well plate in ∼3 min (63, 64). AF488 donor fluorescence was excited with a 473 nm microchip laser from Concepts Research Corporation, and the emission signal was split into two channels by a dichroic mirror and acquired with 517 ± 10 nm (channel 1) and 535 ± 7 nm band-pass filters (channel 2) (Semrock). This instrument uses a unique direct waveform recording technology that enables high-throughput FRET detection at high precision. Fluorescence spectra of each assay plate were also recorded with a SpectraMax Gemini EM plate reader (Molecular Devices). FLT waveforms for each well of 1536-well plates were fit based on a one-exponential decay function using least-squares minimization global-analysis software. The FRET efficiency (E) was determined as the fractional decrease of donor FLT (τ_D_), due to the presence of acceptor (τ_DA_), using the following equation:

(1) $$\text{E}=1-\;\frac{{\text{τ}}_{\text{DA}}}{{\text{τ}}_{\text{D}}}$$

HTS assay quality was calculated based on the effect of positive control (10 μM compound) relative to negative control (0.1% DMSO) and measurement precision, as indexed by the Z′ factor (37):

(2) $$Z^{\text{′}}=1-3\left(\frac{\sigma_{+}+\sigma_{-}}{\left|{\text{μ}}_{+}-{\text{μ}}_{-}\right|}\right)$$

where σ_+_ and σ_−_ are the SDs of the positive and negative control τ_DA_, respectively; μ_+_ and μ_−_ are the means of the positive and negative control τ_DA_, respectively. A value of Z′ ≥ 0.5 indicates an excellent HTS assay, while 0.5>Z′ ≥ 0 indicates an adequate assay (37).

### Surface plasmon resonance (SPR) analysis of hit interaction with the isolated NTR

The SPR experiments were performed using a BIACORE S200 (GE Healthcare) equipped with a NTA sensor chip. NTR with His and MBP tags (>90% pure based on SDS-PAGE) was immobilized using affinity of the His tag to the sensor surface. The surfaces of the sensor chip were activated with 0.5 mM Ni^2+^ solution at a flow rate of 10 μl/min. The NTR at a concentration of 30 μg/ml in 10 mM HEPES, 150 mM KCl, pH 7.4, was immobilized on flow cell 2; flow cell 1 was left blank to serve as a reference surface. To collect kinetic binding data, the hits in the running buffer (10 mM HEPES, 150 mM NaCl, 0.05% P20, pH 7.4, 1% DMSO) were injected over the two flow cells at concentrations of 1.5625, 3.125, 6.25, 12.5, 25, 50, and 100 μM at a flow rate of 30 μl/min and at a temperature of 25 °C. The complex was allowed to associate and dissociate for 60 and 60 s, respectively. The surfaces were regenerated with a 120 s injection of 350 mM EDTA. Data were collected at a rate of 1 Hz. The data were fit using the global data analysis option available within Biacore S200 Evaluation 1.0 software.

### Isolation of sarcoplasmic reticulum vesicles

Crude sarcoplasmic reticulum membrane vesicles were isolated from porcine longissimus dorsi muscle and porcine cardiac left ventricle tissue by differential centrifugation of homogenized tissue, as established previously (65). Skeletal heavy SR (HSR) vesicles, which are enriched with RyR1, were isolated by fractionation of crude skeletal SR vesicles using a discontinuous sucrose gradient, in accordance with our published work (65). All vesicles were flash-frozen and stored at −80 °C.

### [^3^H] ryanodine binding to SR vesicles and data analysis

In 96-well plates, skeletal SR membranes (HSR, 1 mg/ml) and cardiac SR membranes (CSR, 3 mg/ml) were preincubated with 1% DMSO or hit compound for 30 min at 22 °C in a solution containing 150 mM KCl, 5 mM GSH, 1 μg/ml Aprotinin/Leupeptin, 1 mM EGTA, and 65 μM or 1.02 mM CaCl_2_ (as determined by MaxChelator to yield 30 nM or 30 μM of free Ca^2+^, respectively), 0.1 mg/ml of BSA, and 20 mM K-PIPES (pH 7.0). Nonspecific [^3^H]ryanodine binding to SR was assessed by addition of 40 μM nonradioactively labeled ryanodine. Maximal [^3^H]ryanodine binding was assessed by addition of 5 mM adenylyl-imidodiphosphate (AMP-PNP), and in the case of CSR, 20 mM caffeine was added. These control samples were each loaded over four wells per plate. Binding of [^3^H]ryanodine (7.5 and 10 nM for CSR and HSR, respectively) was determined following a 3 h incubation at 37 °C and filtration through grade GF/B glass microfiber filters (Brandel Inc) using a Brandel Harvester. In 4 ml of Ecolite scintillation cocktail (MP biomedicals), [^3^H]ryanodine retained on the filter was counted using a Beckman LS6000 scintillation counter.

### Muscle preparation for single-fiber imaging

All experimental methods using rodents were approved by the Animal Ethics Committees at The University of Queensland and were performed in accordance with the relevant guidelines and regulations. Five-month-old Wistar rats (UQ Biological Resources) were euthanized by asphyxiation *via* CO_2_ exposure, and the *extensor digitorum longus* (EDL) was rapidly excised. Once dissected, muscles were pinned to Sylgard set in a Petri dish containing paraffin oil.

### Confocal imaging for RyR1 leak in skeletal muscle fibers

Bundles of fibers were isolated and exposed to an internal solution containing rhod-5N, as described previously (47). After 15 min incubation, single skeletal muscle fibers were mechanically skinned and mounted in a custom-made chamber. Mounted skinned fibers were imaged using an Olympus FV1000 confocal microscope equipped with an Olympus 0.9NA 40× Plan-Apochromat objective. Rhod-5N was excited with 543-nm HeNe laser and the emission was filtered using the Olympus spectra detector. For tracking Ca^2+^ movements across the t-system membrane, images were continuously recorded in xyt mode with an aspect ratio of 256 × 512, with the long aspect of the image parallel with that of the preparation. Temporal resolution of imaging in this mode where the fluorescence signal was within the borders of the fiber was 0.8 s.

T-system rhod-5N fluorescence was converted to [Ca^2+^]_t-sys_ as previously described (47). To determine the effect of RyR modulators on the RyR Ca^2+^ leak, isolated mechanically skinned fibers were continuously imaged as described above, to obtain a record of t-system rhod-5N fluorescence changes over time as the cytoplasmic solution bathing the fiber was changed. SR and t-system Ca^2+^ was released by bathing the fiber in a solution where free [Mg^2+^] was lowered from 1 to 0.01 mM in the presence of 30 mM caffeine (“release solution”). This chronically opened the RyR, thus thoroughly depleting the SR of Ca^2+^ and consequent depletion of t-system Ca^2+^ occurred *via* the activation of SOCE. Alternatively, the application of 200 nM [Ca^2+^]_cyto_ standard internal solution allowed the uptake of Ca^2+^ and the [Ca^2+^]_t-sys_ reached a steady state that was partially dependent on Ca^2+^ leaking through RyRs into the tight junctional space between the t-system and SR membranes (46). By blocking the RyR with tetracaine (1 mM), it was possible to separate the influence of RyR Ca^2+^ leak on t-system Ca^2+^ steady-state from the level determined by the lower concentration of bulk cytoplasmic Ca^2+^ that could otherwise enter the junctional space. This difference provides a measure of RyR Ca^2+^ leak or a reference for Ca^2+^ leak that we can use to assess the effectiveness of RyR modulators in muscle fibers. FA and tetracaine were dissolved as stock solutions in DMSO (<0.1% DMSO). FA (10 μM), tetracaine (1 mM), and DMSO control (0.1%) were added to the standard internal solution bathing the skinned fibers, at known concentrations, to assess their influence on RyR Ca^2+^ leak.

### Electrically evoked Ca^2+^ transients in rat skeletal muscle fibers

Mechanically skinned fibers were isolated, mounted in a custom chamber, and bathed in a resting physiological solution containing rhod-2, as previously described (21). The Ca^2+^-sensitive dye rhod-2 was used to track cytosolic Ca^2+^ transients when exposed to electrical stimulation. Field pulses at 1 Hz frequency and 4 ms in duration were applied across platinum electrodes parallel to the long axis of the fiber. Imaging for electrical stimulation experiments was in xt mode, at a rate of 2 ms.line^−1^. DMSO vehicle (0.01%), FA (10 μM), and tetracaine were added to the physiological solution. Each treatment was applied precisely at the seventh successive Ca^2+^ transient.

### Confocal imaging of intracellular Ca^2+^ activity in cardiomyocytes

Mouse ventricular cardiomyocytes were isolated enzymatically from hearts of male C57BL/6J mouse (10–12 weeks old, Jackson Laboratory) as previously described with minor modifications (66). Briefly, mice were injected with heparin (500 U/kg body weight) and anesthetized with isoflurane (2 to 5%) to a verified deep surgical plane, whereupon the heart was excised, resulting in euthanasia by exsanguination. Hearts were excised and retrogradely perfused with constant flow on a Langendorff apparatus (37 °C) with Ca^2+^-free normal Tyrode’s solution, gassed with 100% O_2_. Collagenase type II (Worthington Biochemical Co) and protease type XIV (Sigma-Aldrich) were used for enzymatic digestion. Ventricular cardiomyocytes were then mechanically dissociated and filtered through a nylon mesh and allowed to sediment in Tyrode’s solution. The [Ca^2+^] of the Tyrode’s solution was then raised to 0.5 mM in stepwise fashion, and cardiomyocytes were kept at room temperature in this solution until use. All animal handling and laboratory procedures were in accordance with the approved protocols of the University of California, Davis Institutional Animal Care and Use Committee conforming to the Guide for the Care and Use of Laboratory Animals published by the US National Institutes of Health (eighth edition, 2011).

Ca^2+^ activity was imaged *via* confocal microscopy (Bio-Rad, Radiance 2100, 40× objective) in the line scan mode (166 lines/s; ex 488 nm, em >505 nm). Ca^2+^ sparks were recorded from ventricular cardiomyocytes permeabilized with saponin (50 μg/ml) for 1 min in internal solution containing (in mM) 10 HEPES, 1 EGTA, 1 free MgCl_2_ (with free [Ca^2+^] adjusted to 50 nM using MaxChelator), 120 K-aspartate, 10 reduced glutathione, 5 ATP, 5 phosphocreatine di-Na, creatine phosphokinase 5 U/ml, and dextran (MW: 40,000) 8% at pH 7.20. Cardiomyocytes were immediately washed with saponin-free internal solution and then superfused with internal solution (50 nM Ca^2+^_free_) containing the Ca^2+^-sensitive indicator, Fluo-4 pentapotassium salt (10 μM, Invitrogen). Ca^2+^ spark parameters were analyzed over a 10 s period from each line scan recording. Ca^2+^ transients and diastolic Ca^2+^ release events were recorded from intact ventricular cardiomyocytes loaded for 30 min at room temperature with Fluo-4 AM (10 μM, Invitrogen) and Pluronic F-127 (0.02%, Invitrogen) followed by wash and de-esterification for 30 min in Tyrode’s solution containing (in mM) NaCl 140, KCl 4, CaCl_2_ 1.8, MgCl_2_ 1, HEPES 5, Na-HEPES 5, and glucose 5.5 at pH 7.40. To evoke Ca^2+^ transients, intact cardiomyocytes were paced at 1 Hz in a field stimulation chamber (Warner Instruments) at room temperature. Ca^2+^ activity was assessed in paired fashion before and after incubating cardiomyocytes with fusidic acid (FA) for 6 min at each concentration. ImageJ was used for image processing and analysis, and Ca^2+^ sparks were analyzed using the SparkMaster plugin (67). Chemical reagents were purchased from Sigma-Aldrich unless indicated otherwise.

Statistical analyses were performed using GraphPad Prism 9. Normality was verified using the D’Agostino & Pearson test. Repeated measures (at different FA concentrations) one-way ANOVA with Geisser–Greenhouse correction followed by the Dunnett’s multiple comparison post-hoc test was used for normally distributed data to determine individual differences in matched responses. The nonparametric Friedman one-way ANOVA with Dunn’s multiple comparison post-hoc test was used for non-normally distributed data to determine individual differences in matched responses. To determine dose-dependent trends in Ca^2+^ spark responses, the one-sample *t* test on the slopes of linear fits from each cell across [FA] on a log-scale was performed, relative to 0. All data were considered statistically significant at the *p* = 0.05 level.

### Confocal imaging of [Ca^2+^]_ER_ load-leak balance in HEK293 cells

The method for measuring of [Ca^2+^]_ER_ load-leak balance in HEK293 cells was previously described (48). Briefly, HEK293 cells were transiently cotransfected with plasmids encoding GFP-hRyR2, mCer-SERCA2a, and R-CEPIA1er cDNAs. After 48 h of protein expression, transfected cells were used for the confocal experiments. Before each measurement, cells were washed with a solution containing (in mM): 150 K-aspartate, 0.25 MgCl_2_, 0.1 EGTA, 10 HEPES, pH 7.2. Then, the cellular plasma membranes were permeabilized with 0.005% saponin dissolved in the experimental solution containing (in mM): K-aspartate 100; KCl 15; KH_2_PO_4_ 5; MgATP 5; EGTA 0.35; CaCl_2_ 0.22; MgCl_2_ 0.75; HEPES 10; dextran (MW: 40,000) 2%, pH 7.2. Free [Ca^2+^] and [Mg^2+^] were set at 100 nM and 1 mM respectively. After 3 min of saponin incubation, the permeabilized cells were perfused with saponin-free experimental solution.

Changes in [Ca^2+^]_ER_ load-leak balance in permeabilized HEK293 cells were monitored using a laser scanning confocal microscope (Radiance 2000 MP, BioRad) equipped with a 40× oil objective lens (N.A. =1.3). R-CEPIA1er was excited using the 543-nm line of a He–Ne laser, and fluorescence was observed at >580 nm. 2D images (512 × 512 pixels) were collected every 5 s at a scanning speed of 3 ms/line for each measurement.

Cells were perfused with the experimental solution for 3 min to ensure a steady [Ca^2+^]_ER_ signal. Then, the indicated drug concentration was applied for another 5 min. Caffeine (10 mM) was used at the end of each measurement to induce a complete depletion of [Ca^2+^]_ER_ and obtain the F_min_ value. F_max_ was determined upon application of 2 μM ionomycin and 10 mM Ca^2+^. Changes in [Ca^2+^]_ER_ were calculated according to [Ca^2+^]_ER_ = (F−F_min_)/(F_max_−F_min_).

### Statistical analysis

Errors are reported as the standard error of the mean (SE), except when noted. Statistical significance was determined by Student’s *t* test or one-way ANOVA followed by Tukey’s post-hoc test, as indicated, where *p* < 0.05 was considered significant. EC_50_ values were derived from the fits to Hill equations.

## Data availability

All data are contained within the manuscript or supporting information.

## Supporting information

This article contains supporting information.

## Conflict of interest

D. D. T. and R. L. C. hold equity in and serve as executive officers for Photonic Pharma LLC. These relationships have been reviewed and managed by the University of Minnesota. Photonic Pharma had no role in this study.
