# Supplementary material for: Cardiac ryanodine receptor N-terminal region biosensors identify novel inhibitors via FRET-based high-throughput screening
Source: J Biol Chem. 2021 Nov 16;298(1):101412. doi: 10.1016/j.jbc.2021.101412 (PMC8689225; doi:10.1016/j.jbc.2021.101412)
Supplement: Figures S1–S8 and Tables S1–S4 [file mmc1.pdf]

Supporting Information:

Cardiac ryanodine receptor N-terminal region biosensors identify novel inhibitors *via* FRET-based high-throughput screening

Jingyan Zhang[1], Daniel P. Singh[2], Christopher Y. Ko[3], Roman Nikolaienko[4], Siobhan M. Wong King Yuen[5], Jacob A. Schwarz[1], Levy M. Treinen[1], Ching-Chieh Tung[5], Kaja Rožman[6], Bengt Svensson[1], Courtney C. Aldrich[6], Aleksey V. Zima[4], David D. Thomas[1], Donald M. Bers[3], Bradley S. Launikonis[2], Filip Van Petegem[5], and Razvan L. Cornea[1]*

From the [1]Department of Biochemistry, Molecular Biology and Biophysics, University of Minnesota, Minneapolis, Minnesota, USA; [2]School of Biomedical Sciences, The University of Queensland, Brisbane, Queensland, Australia; [3]Department of Pharmacology, University of California, Davis, California, USA; [4]Department of Cell and Molecular Physiology, Loyola University Chicago, Chicago, Illinois, USA; [5]Department of Biochemistry and Molecular Biology, Life Sciences Institute, University of British Columbia, Vancouver, British Columbia, Canada; [6]Department of Medicinal Chemistry, University of Minnesota, Minneapolis, Minnesota, USA

* For correspondence: Razvan L. Cornea, corne002@umn.edu.

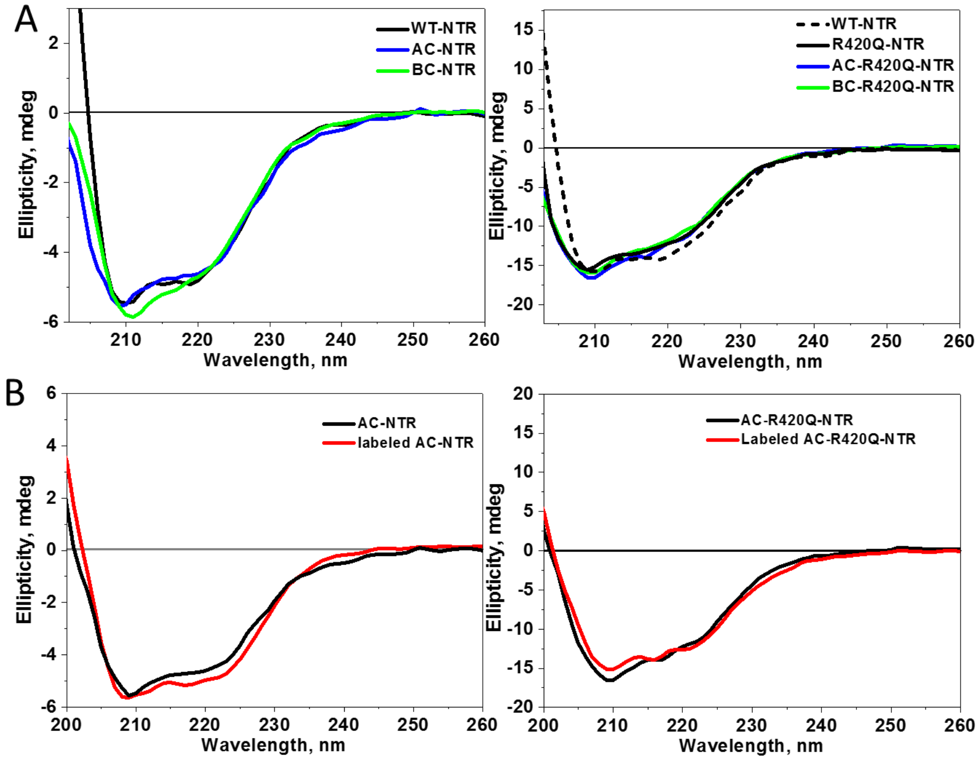

Figure S1. A) CD spectra of the WT-NTR, Cys-light AC- and BC-NTR, and the mutant R420Q, Cys-light AC-R420Q and BC-R420Q. B) CD spectra of the labeled and unlabeled AC-NTR and AC-R420Q-NTR.

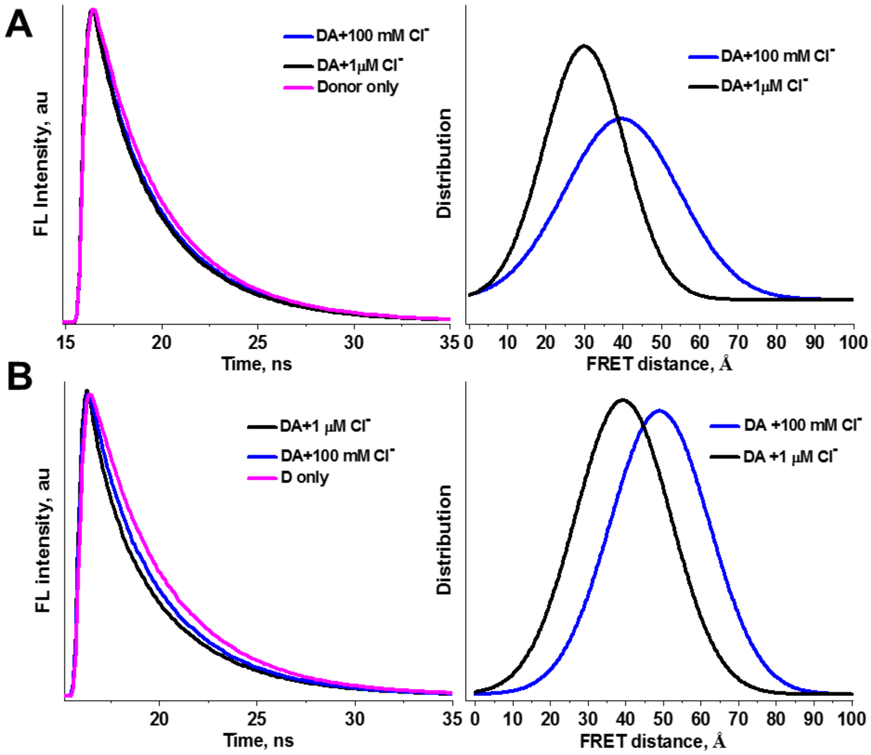

Figure S2. Representative fluorescence decay waveforms and inter-probe FRET distance distributions of the donor labeled and donor-acceptor labeled AC-NTR-RyR2 A) and BC-NTR-RyR2 B) constructs in the presence of 0.001 and 100 mM [Cl^-].

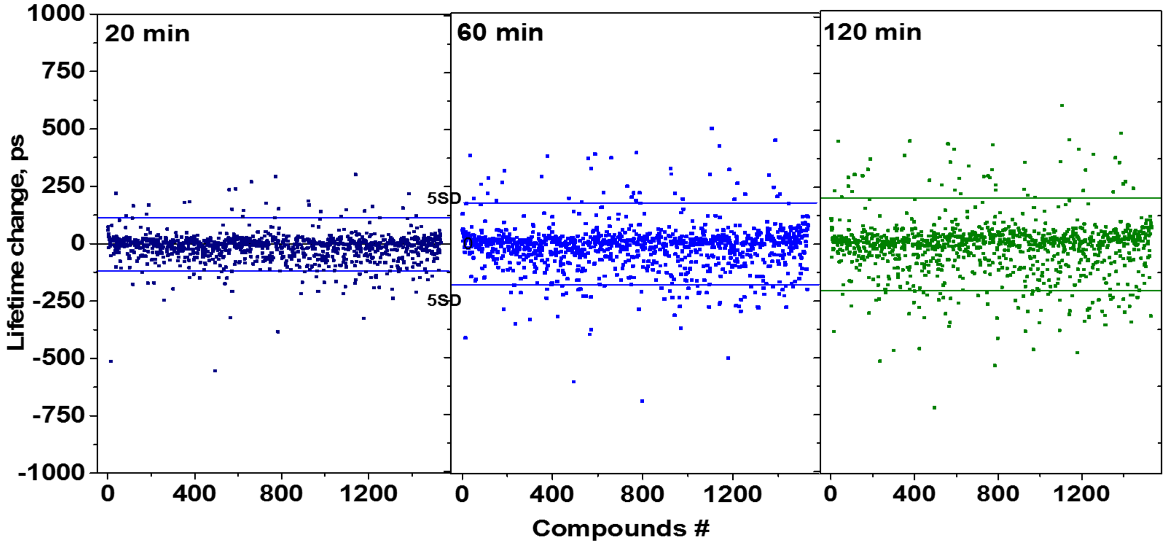

Figure S3. HTS results with AC-NTR-RyR2 biosensor obtained after 20, 60, and 120 min of incubation with 1280 compounds in 1536-well assay plates. Solid lines are ± 5SD in each panel.

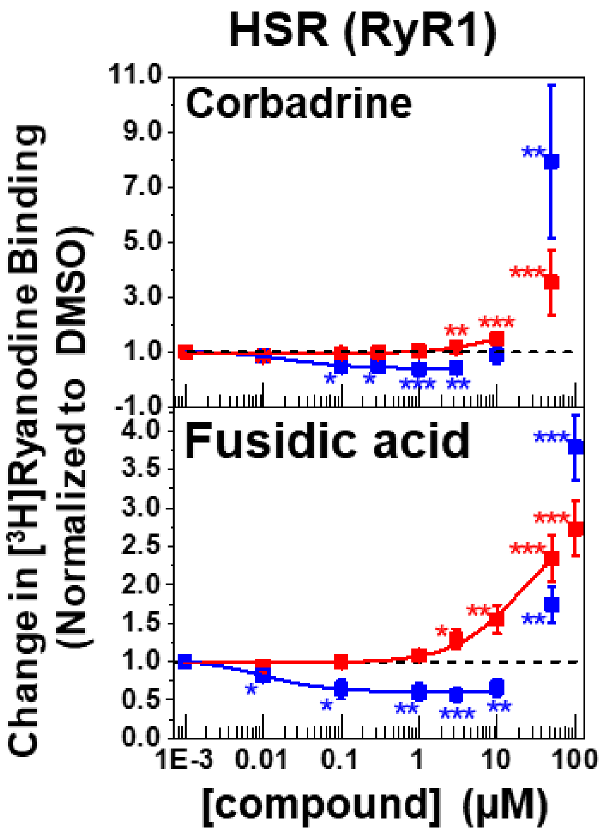

Figure S4. Dose-dependent effect of the corbadrine (top) and fusidic acid (bottom) on [^{3}H]ryanodine binding to porcine skeletal heavy SR (HSR) at 30 nM (blue) and 30 μM (red) free [Ca^{2+}]. Results are shown normalized relative to the values for no-drug control (DMSO), respectively, mean ± SD, $n \geq 4$. The asterisks (*,**,***) indicate the level of significance in the difference from DMSO controls using unpaired Student's t-test: ***$P \leq 0.001$ (extremely significant), $0.001 \leq$ **$P < 0.01$ (very significant), $0.01 \leq$ *$P < 0.05$ (significant), no-asterisk $P \geq 0.05$ (statistically insignificant difference relative to the DMSO control).

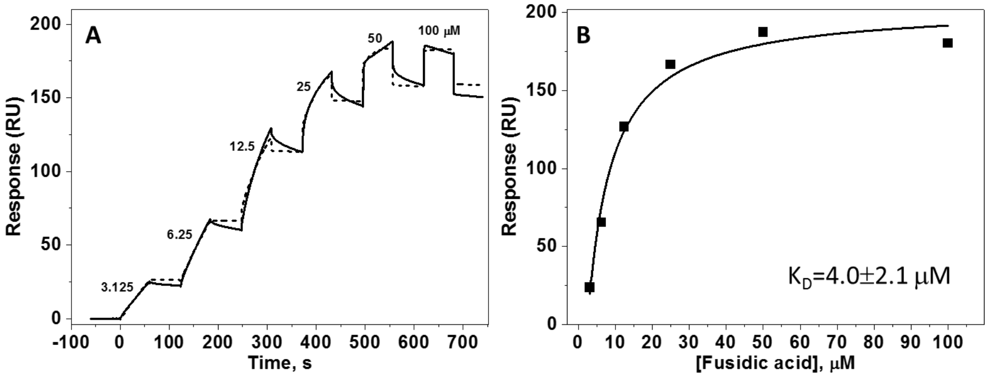

Figure S5. A) Single-cycle kinetic analyses of fusidic acid (FA) and the NTR interactions using surface plasmon resonance. Representative solid trace depicts the responses from the NTR surface in the presence of different concentration of FA. Unfortunately, repeated measurements are difficult to obtain because the NTR tends to stick on the sensor chip during the regeneration step. The trace was fitted with a heterogenous model using global data analysis (dotted line), resulting in one component with micromolar K_D and another component with nanomolar K_D. FA concentrations are denoted on the sensorgram. B) Plot of the response (RU) vs. FA concentrations in A), and the Hill fit (solid line) of the responses.

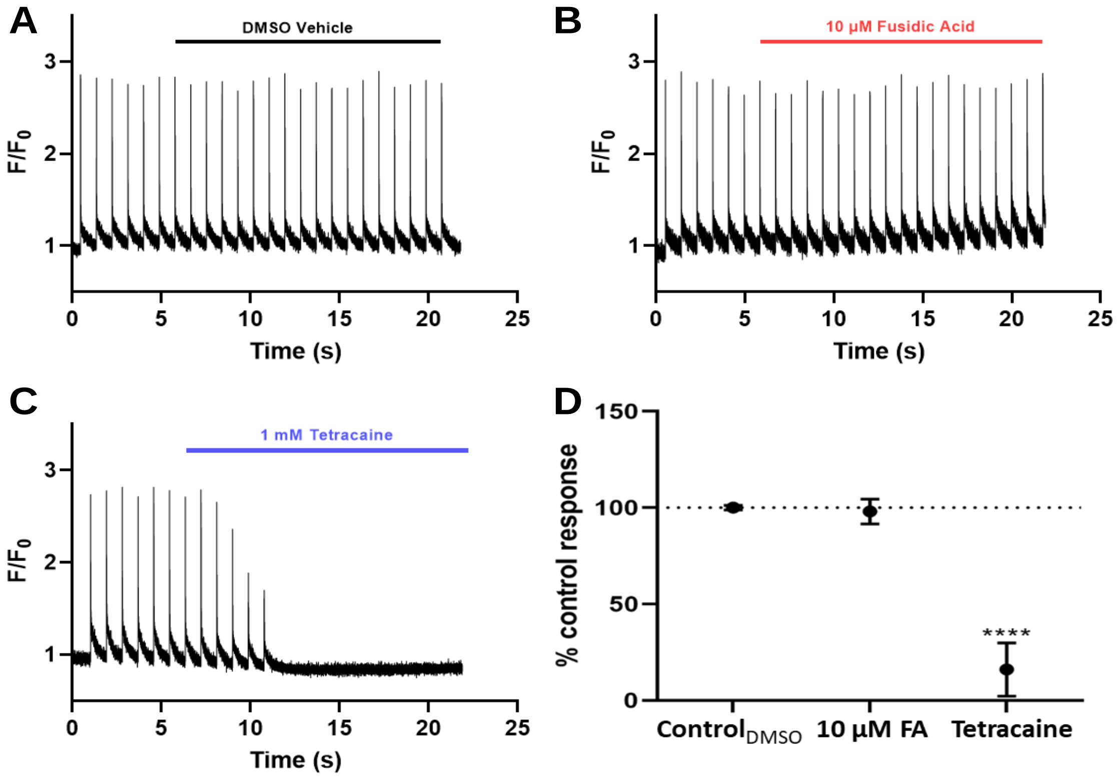

Figure S6. Effect of fusidic acid (FA) on electrically evoked Ca^{2+} transients. In rat skinned fibers, cytosolic Ca^{2+} transients were obtained by confocal line scans parallel to the fiber long axis, with corresponding line averaged and normalized rhod-2 fluorescence signals (F/F_0). Ca^{2+} transients were elicited by electrical field stimulation at 1 Hz. Representative traces are shown for Ca^{2+} transients before and after (indicated by bar) the addition of A) DMSO (vehicle), B) 10 µM FA or C) 1 mM tetracaine. D) Ca^{2+} transient data (% of control response) is presented as means ± SD, ****$P < 0.0001$ vs. vehicle, n = 6 – 12 individual fibers.

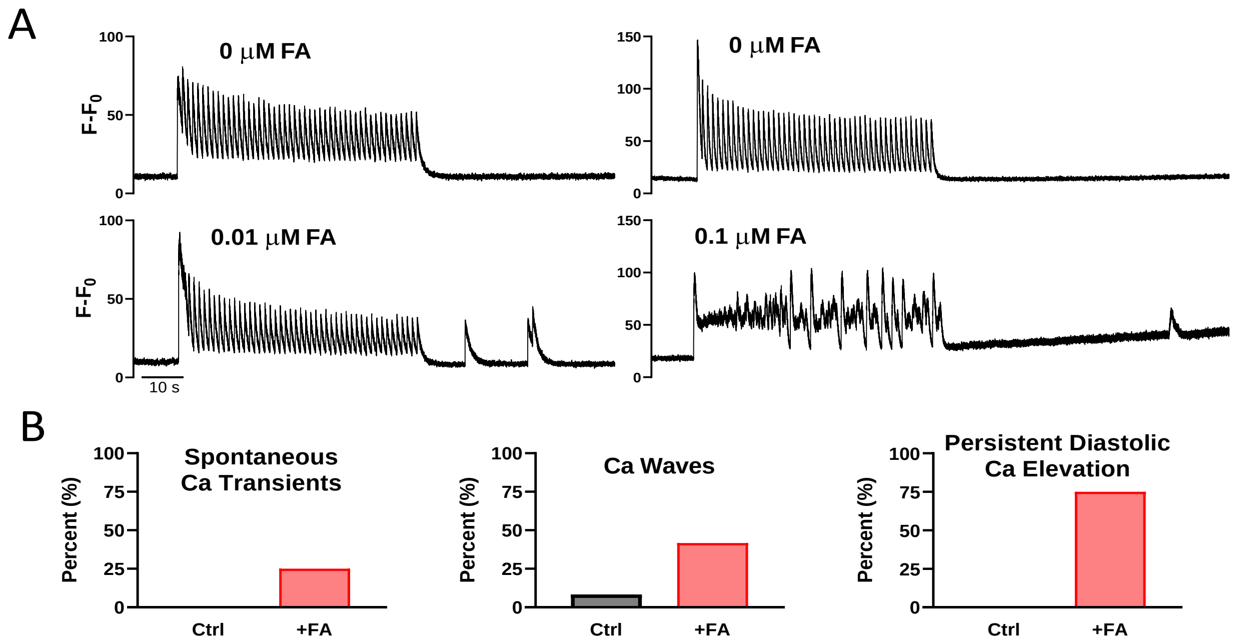

Figure S7. A) Representative Ca^{2+} transient responses to low-dose (0.01 and 0.1 µM) fusidic acid (FA) exposures (6 min) in intact mouse ventricular cardiomyocytes paced at 1 Hz for 50 s. B) Proportion of 12 paced intact myocytes exhibiting arrhythmogenic Ca^{2+} activity before and after exposure (6 min) to FA across all concentrations (0.01 to 10 µM). $n_{cell}/N_{animal} = 12/4$.

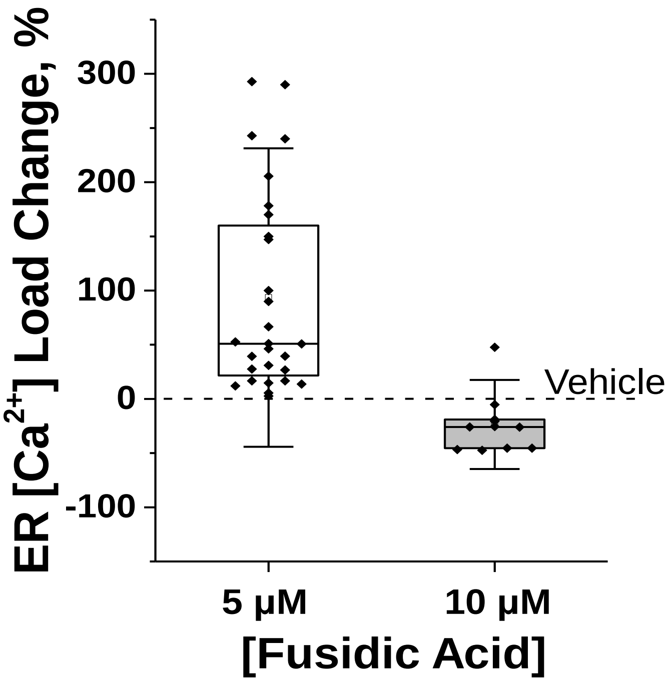

Figure S8. The addition of 5 µM of fusidic acid (FA) caused a $[Ca^{2+}]_{ER}$ increase of 93.56 ± 17.35% compared to vehicle ($P < 0.001$, N = 28 cells). The increase of FA concentration to 10 µM caused a $[Ca^{2+}]_{ER}$ depletion of 23.6 ± 8.26% compared to vehicle ($P < 0.05$, N = 11 cells). Data are presented with the box at 25-75 percentile and error bars at 1.5SD. P-values were obtained from paired-sample Student's t-tests for each concentration of FA.

Table S1. Distance (R) and full-width at half maximum (FWHM) from fitting of FLT-detected intramolecular FRET between donor-acceptor probes in the AC- and BC-NTR biosensors corresponding to WT- and R420Q-NTR, in the presence of high (100 mM) and low (0.001 mM) [Cl^-]. SDs were calculated from the average of three experiments.

**[Cl^-] (mM)**	**BC-NTR**		**AC-NTR**		**BC-R420Q-NTR**	
	R (Å)	FWHM (Å)	R (Å)	FWHM (Å)	R (Å)	FWHM (Å)
**0.001**	40.1±1.0	35.2±3.3	30.8±0.7	25.8±8.3	49.6±6.3	49.0±7.1
**100**	51.7±5.4	35.7±17.5	38.9±1.4	37.7±1.9	49.5±6.4	40.0±21.2

Table S2. Reproducibility of hits obtained via three HTS runs using the AC- and BC-NTR constructs, after several incubation times with library compounds.

**Incubation time (min)**	***n* screens (out of 3)**	**Number of hits (Hit rate)**		
		**AC-NTR**	**BC-NTR**	**AC-&BC-NTR**
20	2	42 (3.2%)	9(0.7%)	4
	3	13 (1.0%)	0	
60	2	72 (5.6%)	30 (2.3%)	10
	3	46 (3.5%)	2 (0.16%)	
120	2	63 (4.9%)	30 (2.3%)	9
	3	50 (3.9%)	4 (0.3%)	

Table S3. Chemical names and abbreviations of the eight hits studied in this work.

**Abbreviation**	**Chemical name**
CPNQ	5-[4-(4-Chlorobenzoyl)-1-piperazinyl]-8-nitroquinoline
oltipraz M2	7-Methyl-6,8-bis(methylthio)-pyrrolo[1,2-a]pyrazine
GANT61	2-[[3-[[2-(dimethylamino)phenyl]methyl]-2-pyridin-4-yl-1,3-diazinan-1-yl]methyl]-N,N-dimethylaniline
corbadrine	4-[(1*R*,2*S*)-2-amino-1-hydroxypropyl]benzene-1,2-diol
fusidic acid	(2*Z*)-2-[(3*R*,4*S*,5*S*,8*S*,9*S*,10*S*,11*R*,13*R*,14*S*,16*S*)-16-acetyloxy-3,11-dihydroxy-4,8,10,14-tetramethyl-2,3,4,5,6,7,9,11,12,13,15,16-dodecahydro-1*H*-cyclopenta[a]phenanthren-17-ylidene]-6-methylhept-5-enoic acid
tacrolimus	(3*S*,4*R*,5*S*,8*R*,9*E*,12*S*,14*S*,15*R*,16*S*,18*R*,19*R*,26a*S*)-5,19-dihydroxy-3-{(1*E*)-1-[(1*R*,3*R*,4*R*)-4-hydroxy-3-methoxycyclohexyl]prop-1-en-2-yl}-14,16-dimethoxy-4,10,12,18-tetramethyl-8-(prop-2-en-1-yl)-5,6,8,11,12,13,14,15,16,17,18,19,24,25,26,26a-hexadecahydro-3*H*-15,19-epoxypyrido[2,1-c][1,4]oxazacyclotricosine-1,7,20,21(4*H*,23*H*)-tetrone
temsirolimus	(1*R*,2*R*,4*S*)-4-{(2*R*)-2-[(3*S*,6*R*,7*E*,9*R*,10*R*,12*R*,14*S*,15*E*,17*E*,19*E*,21*S*,23*S*,26*R*,27*R*,34a*S*)-9,27-dihydroxy-10,21-dimethoxy-6,8,12,14,20,26-hexamethyl-1,5,11,28,29-pentaoxo-1,4,5,6,9,10,11,12,13,14,21,22,23,24,25,26,27,28,29,31,32,33,34,34a-tetracosahydro-3H-23,27-epoxypyrido[2,1-c][1,4]oxazacyclohentriacontin-3-yl]propyl}-2-methoxycyclohexyl 3-hydroxy-2-(hydroxymethyl)-2-methylpropanoate
Ro 90-7501	2'-(4-Aminophenyl)-1H,1'H-2,5'-bibenzimidazol-5-amine

Table S4. EC_{50} values of the five hits obtained from the FRET dose-response and [^{3}H]Ryanodine binding assay. Mean ± SE were obtained from the fit using the Hill function. EC_{50} is in μM. The confidence level (CL) is set at 90%.

Hit	FRET dose-response				[^{3}H]Ryanodine binding assay															
					Skeletal HSR								Cardiac SR							
					[Ca^{2+}], 30 nM				[Ca^{2+}], 30 μM				[Ca^{2+}], 30 nM				[Ca^{2+}], 30 μM			
	EC_{50}	SE	LCL	HCL	EC_{50}	SE	LCL	HCL	EC_{50}	SE	LCL	HCL	EC_{50}	SE	LCL	HCL	EC_{50}	SE	LCL	HCL
**CPNQ**	1.3	0.5	0.4	2.3	0.02	0.02	-0.03	0.07	-	-	-	-	0.02	0.02	-0.03	0.07	-	-	-	-
**Oltipraz M2**	21.7	17.3	-6.5	43.7	0.005	0.003	-0.001	0.01	-	-	-	-	-	-	-	-	-	-	-	-
**GANT61**	15.8	8.6	-1.5	33.1	0.004	0.004	-	-	9.4	6.5	-	-	0.004	0.006	-0.01	0.02	17.3	7.7	1.8	32.8
**Corbadrine**	3.6	0.6	2.4	4.8	0.02	0.01	-0.004	0.04	15.3	19.2	-25	56	0.01	0.01	-0.02	0.03	-	-	-	-
**Fusidic acid**	3.8	0.5	2.9	4.8	0.01	0.005	-0.003	0.02	21.1	5.4	9.5	32.6	1.7	1.1	-0.7	4.1	-	-	-	-

*EC_{50}, half maximal effective concentration; SE, standard error; LCL, low confidence limit; HCL, high confidence limit.
